# Cell class-specific modulation of attentional signals by acetylcholine in macaque frontal eye field

**DOI:** 10.1073/pnas.1905413116

**Published:** 2019-09-16

**Authors:** Miguel Dasilva, Christian Brandt, Sascha Gotthardt, Marc Alwin Gieselmann, Claudia Distler, Alexander Thiele

**Affiliations:** ^a^Institute of Neuroscience, Newcastle University, Newcastle upon Tyne, NE2 4HH, United Kingdom;; ^b^Allgemeine Zoologie und Neurobiologie, Ruhr-Universität Bochum, 44801 Bochum, Germany

**Keywords:** attention, acetylcholine, frontal cortex

## Abstract

Attention is critical to high-level cognition, and attentional deficits are a hallmark of cognitive dysfunction. A key transmitter for attentional control is acetylcholine, but its cellular actions in attention-controlling areas remain poorly understood. Here we delineate how muscarinic and nicotinic receptors affect basic neuronal excitability and attentional control signals in different cell types in macaque frontal eye field. We found that broad spiking and narrow spiking cells both require muscarinic and nicotinic receptors for normal excitability, thereby affecting ongoing or stimulus-driven activity. Attentional control signals depended on muscarinic, not nicotinic receptors in broad spiking cells, while they depended on both muscarinic and nicotinic receptors in narrow spiking cells. Cluster analysis revealed that muscarinic and nicotinic effects on attentional control signals were highly selective even for different subclasses of narrow spiking cells and of broad spiking cells. These results demonstrate that cholinergic receptors are critical to establish attentional control signals in the frontal eye field in a cell type-specific manner.

## Significance

Attention improves perceptual abilities by modulating sensory processing. A key transmitter for attentional control is the neurotransmitter acetylcholine. We show that acetylcholine promotes attentional signals in frontal cortex by differentially activating 2 cholinergic receptor types in different cell groups. Acetylcholine promotes attentional control signals through muscarinic receptors in specific subclasses of broad spiking cells. Moreover, it promotes attentional control signals through muscarinic and nicotinic receptor activation in specific subclasses of narrow spiking cells. Thus, attentional control signals in the frontal eye field (FEF) are supported through nicotinic and muscarinic receptor activation in a highly cell type-specific manner.

Acetylcholine (ACh) is a key neuromodulator critical to high-level cognitive functions, such as learning, memory, and attention (1–3). Cholinergic deficits are a hallmark of attentional dysfunctions associated with Alzheimer disease, and drugs that enhance ACh levels in the brain are used to alleviate some of the associated symptoms (4). Cellular signatures of attention in sensory and high-level association areas are well studied, but their neuropharmacological underpinnings remain poorly understood. Cellular signatures of attention include increased firing rates (5, 6), reduced rate variability (7–9), reduced noise correlation (8–11), and altered oscillatory activity (12–14). These modulations are driven by cortico-cortical feedback connections (15, 16), and one of the key areas mediating these effects is the frontal eye field (FEF) (14–19).

Attention-induced rate changes in primary visual cortex (V1) are dependent on muscarinic, not nicotinic ACh receptors (20), while alterations of attention-induced changes to rate variability and pairwise noise correlations depend on *N*-methyl-d-aspartate (NMDA) receptors (8). Clinical and behavioral studies suggest that the main attention-related benefits from cholinergic enhancement are due to increased activity not in sensory areas, but in frontal and parietal association areas (21, 22), through muscarinic and nicotinic receptors (23–25). Thus, the selective involvement of muscarinic receptors to attentional modulation of firing rates in primary visual cortex may be an anomaly, caused by the unique expression pattern of cholinergic receptors in macaque V1, where muscarinic receptors occur predominantly on gamma-aminobutyric acid (GABA)ergic cells, while in extrastriate cortex they also occur more frequently on pyramidal cells (26–28), possibly in combination with variations in modulatory compartments, such as subcellular receptor expression differences, differences in degradation pathways, or tissue tortuosity across cortex (29). Based on the differences in cell type-specific expression of cholinergic receptors (26, 27, 30–32) we hypothesized that muscarinic and nicotinic receptors contribute to neuronal signatures of attention in frontal cortex, possibly in a cell type-dependent manner. To investigate this, we recorded from FEF cells and performed pharmacological analysis of different ACh receptors by means of iontophoresis, while monkeys performed a covert sustained spatial attention task. We found that broad and narrow spiking cells required nicotinic and muscarinic receptor activation to support normal excitability (neuronal gain control). However, attentional rate modulation in broad spiking FEF cells depended exclusively on muscarinic receptor activation, while in narrow spiking FEF cells it depended on muscarinic and nicotinic receptor activation. Cluster analysis revealed that muscarinic receptors contributed to attentional modulation only in specific subgroups of broad spiking cells. Equally, muscarinic and nicotinic receptors contributed to attentional modulation only in specific subgroups of narrow spiking cells. Our data reveal a cell type-specific contribution of cholinergic receptors to attentional signals in primate frontal cortex.

## Results

**Fig. 1.**
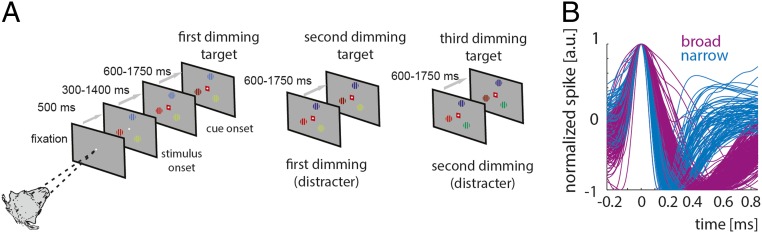
(*A* and *B*) Cartoon of the task (*A*) and recorded spike waveforms (*B*). (*A*) Monkeys fixated centrally. Five hundred milliseconds after fixation onset 3 colored gratings were presented equidistant from the fixation spot. One of the gratings was placed in the receptive field of the neuron under study. After a variable time (300 to 1,400 ms) a central colored cue indicated which stimulus was behaviorally relevant on the current trial. The animal had to covertly monitor this stimulus and wait for it to change luminance (referred to as target dimming). The target dimming could occur first, second, or third in the sequence of dimming events (*Left* to *Right*). Distracter dimming had to be ignored by the monkey. Detection of target dimming was indicated by releasing a hand-held touch bar. (For additional details see *Methods*). (*B*) Normalized spike average waveforms of all narrow (turquoise) and all broad (purple) spiking cells recorded.

We recorded from 344 FEF cells from 2 monkeys, performing a covert top–down spatial attention task under control conditions and with ACh, scopolamine (Scop, muscarinic antagonist), or mecamylamine (Mec, nicotinic antagonist) iontophoretically applied. Animals had to fixate centrally on a computer screen, while holding a touch bar. Following successful initial fixation, 3 differently colored stimuli occurred on the screen, 1 centered on the neuron’s receptive field (RF). After a variable time, a color cue, displayed at fixation, indicated to the monkey which stimulus to attend to. The animal had to monitor this stimulus for luminance decrements (“dimming”) and ignore dimming of the other stimuli. When the cued stimulus dimmed, the animal had to release the touch bar to receive a fluid reward (Fig. 1*A*, *Methods*, *SI Appendix*, and ref. 33). With 3 potential dimming times, the animals had to monitor the cued stimulus throughout the trial. Hit rates (*SI Appendix*) were 0.997 on average; i.e., animals missed fewer than 1/200 target dimmings (chance-level hit rate = 0.33). Correct rejection rates (unreported distractor dimmings, *SI Appendix*) were 0.959; i.e., fewer than 1/20 distractor dimmings were reported. This corresponds to d-prime values of >4.3 for all stimulus locations. Thus, the animals performed the task as intended, heeding the cue, ignoring irrelevant dimmings and reporting target dimmings reliably (*SI Appendix*, Fig. S1).

**Fig. 2.**
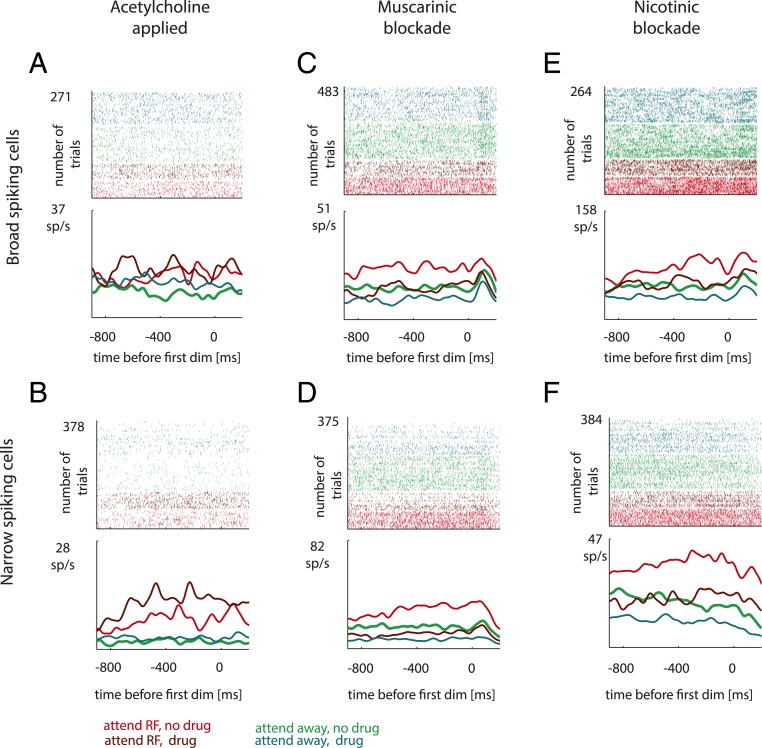
(*A–F*) Single-cell examples of cholinergic modulation of firing rates and of attentional effects for broad (*A*, *C*, and *E*) and narrow (*B*, *D*, and *F*) spiking cells, when acetylcholine, scopolamine, or mecamylamine was applied. Shown are raster plots and peri-stimulus time histograms relative to the time of the first dimming, separately for the 2 attention and 2 drug conditions (color coded). Attention to the receptive field, no drug condition, light red; attention away from the receptive field, no drug condition, light green; attention to the receptive field, drug condition, dark red; and attention away from the receptive field, drug condition, dark green.

For each cell we determined whether neuronal activity was affected by attention (factor 1) or drug application (factor 2) or whether there was an interaction between factors, using a 2-factor ANOVA. The time period analyzed was from −500 ms to 0 ms before the time of the first dimming, which is the time period where the difference between attend RF and attend away conditions is maximal (single-cell examples, Fig. 2; population data, *SI Appendix*, Fig. S6). Of 344 cells, 258 were broad spiking cells (action potential peak-to-trough time [P2T] > 250 μs), and 86 were narrow spiking cells (action potential P2T ≤ 250 μs). Fig. 1*B* shows associated spike waveforms. Sixty-five of 86 (75.4%) cells tested with ACh showed differential activity for attention conditions, 68/86 (79.1%) cells were affected by drug application, and 56/86 (65.1%) cells were affected by both. One hundred sixteen of 136 (85.3%) cells tested with Scop showed differential activity for attention conditions, 86/136 (63.2%) cells were affected by drug application, and 74/136 (54.4%) cells were affected by both. One hundred four of 122 (85.2%) cells tested with Mec showed differential activity for attention conditions, 80/122 (65.5%) cells were affected by drug application, and 68/122 (55.7%) cells were affected by both (*SI Appendix*, Table S1).

Fig. 2 shows example attention and drug effects on neuronal activity. Significance of activity differences was assessed by comparing single-trial activity for different attention and drug conditions, using a 2-factor ANOVA (*F* and *P* values reported below). To obtain a single measure of attentional modulation we calculated the area under the receiver operating characteristic curve (AUROC, *Methods*; separately for drug off and drug on), which indicates how well an ideal observer can decode the locus of attention from single-trial firing rates, and we calculated Cohen’s D (*Methods*).

### ACh Effects on Example Cells.

Fig. 2*A* shows raster plots and peri-stimulus time histograms of an example broad spiking cell (P2T = 415 μs) with and without ACh applied. Attending to the RF resulted in a significantly higher firing rate than attending away (*F*_1,270_ = 63.83, *P* < 0.001). ACh application increased activity (*F*_1,270_ = 11.82, *P* = 0.001), but it reduced attentional modulation (significant attention × drug interaction: *F*_1,270_ = 7.46, *P* = 0.007, AUROC_no_ _drug_ = 0.85, AUROC_drug_ = 0.67). Fig. 2*B* shows effects of ACh application on a narrow spiking cell (P2T = 210 μs). Attention increased firing rates (*F*_1,377_ = 251.1, *P* < 0.001), drug application increased firing rates (*F*_1,377_ = 36.72, *P* < 0.001), and, atypically, it increased attentional modulation (attention × drug interaction: *F*_1,599_ = 23.92, *P* < 0.001, AUROC_no_ _drug_ = 0.84, AUROC_drug_ = 0.93).

### Effect of Muscarinic Blockade on Example Cells.

Fig. 2*C* shows effects of attention and muscarinic blockade on a broad spiking cell (P2T = 409 μs). Attention to the receptive field increased firing rates (*F*_1,479_ = 85.65, *P* < 0.001). Muscarinic blockade reduced activity (*F*_1,479_ = 82.41, *P* < 0.001), and it reduced attentional modulation (attention × drug interaction: *F*_1,479_ = 9.82, *P* = 0.002, AUROC_no_ _drug_ = 0.79, AUROC_drug_ = 0.69). Similar results occurred for the narrow spiking cell (P2T = 194 μs) in Fig. 2*D*. Attention to the receptive field increased firing rates (*F*_1,371_ = 88.07, *P* < 0.001). Muscarinic blockade reduced activity (*F*_1,371_ = 177.1, *P* < 0.001), and it reduced attentional modulation (attention × drug interaction: *F*_1,371_ = 23.34, *P* < 0.001, AUROC_no_ _drug_ = 0.83, AUROC_drug_ = 0.63).

### Effect of Nicotinic Blockade on Example Cells.

Fig. 2*E* shows effects of attention and nicotinic blockade on a broad spiking cell (P2T = 275 μs). Attention to the receptive field increased firing rates (*F*_1,263_ = 135.7, *P* < 0.001). Nicotinic blockade reduced activity (*F*_1,263_ = 85.82, *P* < 0.001), and it reduced attentional modulation (attention × drug interaction: *F*_1,263_ = 5.02, *P* = 0.026, AUROC_no_ _drug_ = 0.88, AUROC_drug_ = 0.85). Fig. 2*F* shows the respective effects on a narrow spiking cell (P2T = 221 μs). Attention to the receptive field increased firing rates (*F*_1,380_ = 175.8, *P* < 0.001). Nicotinic blockade reduced activity (*F*_1,380_ = 100.6, *P* < 0.001), and it reduced attentional modulation (attention × drug interaction: *F*_1,380_ = 14.41, *P* < 0.001, AUROC_no_ _drug_ = 0.91, AUROC_drug_ = 0.71).

### Subdividing Broad and Narrow Cell Types.

So far we have subdivided cells based on spike waveform width, in line with many primate studies (e.g., refs. 7, 34, and 35), often assuming that narrow spiking cells are (largely) inhibitory fast spiking interneurons, while broad spiking cells are largely pyramidal cells. However, almost all pyramidal cells in macaque motor cortex M1 express Kv3.1b potassium channels and have the potential to be narrow spiking (36). Moreover, a large majority of macaque prefrontal cortex inhibitory interneurons are not parvalbumin-positive fast spiking neurons and have broad spiking waveforms (37–41). Finally, calretinin- and calbindin-positive fast spiking interneurons exist (39). Thus, the narrow–broad divide benefits from further subdivision, with additional physiological characteristics taken into account. To do so, we performed cluster analysis (42). For cluster identification we used P2T, coefficient of variation (CV) of the interspike interval (ISI), coefficient of variation of neighboring ISIs (CV2), local variation (Lv) of the ISI, firing rate, variability of firing rate (Fano factor [FF]), and strength of attentional modulation (AUROC) (details in *Methods* and *SI Appendix*). We included parameters that together explained at least 90% of the variance (*SI Appendix* and ref. 42). This left P2T, CV2, Lv, FR, and AUROC as clustering parameters. Including AUROC as a clustering parameter means that clustering was performed on physiological and functional properties. We believe this is justified, as our aim was to determine whether cholinergic drugs differently affect different cell types (including dissociating between cells more and less strongly affected by attention). We used the Akaike information criterion (AIC) and the Bayesian information criterion (BIC) to select the appropriate number of clusters. AIC and BIC allow model selection based on how well a model fits the data, relative to alternative models. We tested *n* = 2, 3, .. to 12 clusters. Based on AIC, data were optimally divided into 7 clusters. Based on BIC data were optimally divided into 6 clusters (*SI Appendix*). Dividing cells into 6 clusters resulted in more mixing of broad and narrow spiking cells within single clusters. This was much reduced using 7 clusters (*SI Appendix*, Fig. S5). Since broad and narrow spiking is a well-established criterion to subdivide cell classes, we used 7 clusters for further analysis. Four of 7 cell clusters contained almost exclusively (a single exception) broad spiking cells (B1 to B4; *SI Appendix*, Fig. S5*A*). Two cell clusters predominantly contained narrow spiking cells (N1 and N2; distribution of waveform width in *SI Appendix*, Fig. S5*A*, *Right*), and 1 cell cluster contained an approximately equal number of narrow and broad spiking cells (N3; *SI Appendix*, Fig. S5*A*). Spike waveform distribution for N3 was not unimodal (calibrated Hartigan’s dip test, false discovery rate [FDR]-adjusted *P* < 0.001). None of the other waveform distributions were significantly bi/multimodal (calibrated Hartigan’s dip test, all FDR-adjusted *P* > 0.1). For details regarding physiological and functional differences between cell clusters see *SI Appendix*, Fig. S6. Next we describe how cholinergic modulation affected cell separation along the dichotomic broad–narrow divide, followed by how it affected clusters B1 to B4 and N1 to N3.

### Drug Effects on General Neuronal Activity.

**Fig. 3.**
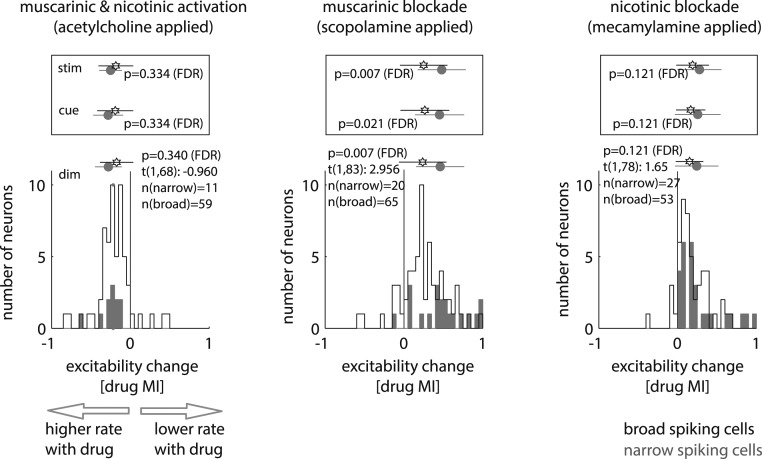
Effect of cholinergic drugs on neuronal excitability, quantified through drug MIs (averaged across attention conditions). Data for broad spiking cells are shown by black outlined histograms and black stars and SEM and those for narrow spiking cells by gray shaded histograms and gray dot and SEM. The 3 different drug conditions are shown separately for the dimming (dim) aligned response period as histograms. Means and SDs are shown as shaded circles and open stars. False discovery rate-corrected *P* values (along with *t* statistics and sample sizes) indicate whether broad and narrow spiking cells were differently affected by the drug application. *Insets* above the histograms show the means and SDs for the period after stimulus onset (stim) and the period after cue onset (cue).

To assess how drug application affected neuronal excitability we compared attend RF as well as attend away firing rates with and without drug applied. We first describe results when separately analyzing broad and narrow spiking cells. We analyzed drug effects with activity aligned to stimulus onset, to cue onset, and to time of first dimming. Cells significantly affected by drug application were included (2-factor ANOVA, main effect of drug or drug × attention interaction; see *SI Appendix*, Table S1 for sample sizes). ACh significantly increased firing rates in both attention conditions (attend RF and attend away), in broad and narrow spiking cells and all 3 analysis periods (all FDR-corrected *P* values <0.001, 2-sided *t* test; for exact numbers and *t* statistics see *SI Appendix*, Table S2). Scop and Mec significantly decreased firing rates (all FDR-corrected *P* values <0.001, 2-sided *t* test; for exact numbers and *t* statistics see *SI Appendix*, Table S2). To quantify effects across attention conditions we calculated a drug modulation index (MI), $\left(Drug\;MI=\frac{rate_{no\;drug}-rate_{drug}}{rate_{no\;drug}+rate_{drug}}\right).$ Drug MI histograms (along with means and SDs) for broad and narrow spiking cells are shown in Fig. 3. ACh application generally increased firing rates, and thus drug MI distributions were on average negative and significantly different from 0 (*P* < 0.001, 2-sided *t* test, both cell types, all response periods, FDR corrected for multiple comparisons). Mean drug MIs of narrow and broad spiking cells were not significantly different (*t*(1, 68) = −0.956, *P* = 0.340, 2-sided *t* test, FDR corrected for multiple comparisons). With Scop applied drug MIs were on average positive and significantly different from 0 (*P* < 0.001, 2-sided *t* test, both cell types, all response periods, FDR corrected for multiple comparisons), reflecting the suppressive action of muscarinic receptor blockade. Narrow spiking cells had significantly larger drug MIs than broad spiking cells (predimming period: *t*(1,83) = 2.956, *P* = 0.007, 2-sided *t* test, FDR corrected for multiple comparisons); i.e., narrow spiking cells were more strongly modulated by muscarinic blockade than broad spiking cells. This difference occurred for all 3 response periods (Fig. 3, *Insets*). The difference in muscarinic blockade efficacy on the 2 cell types was not a consequence of differential firing rates between narrow and broad spiking cells (*SI Appendix*). Nicotinic blockade equally resulted in positive drug MIs for both cell types (*P* < 0.001, 2-sided *t* test, both cell types, all response periods, FDR corrected for multiple comparisons), but there was no significant difference of drug MIs between the 2 cell types (*t*(1,78) = 1.65, *P* = 0.121, FDR corrected for multiple comparisons, 2-sided *t* test). Thus, nicotinic blockade reduced the excitability in narrow and in broad spiking cells similarly.

We can exclude the possibility that differing levels of drug application account for the drug results on firing rates or on attentional modulation in narrow vs. broad spiking cells (details *SI Appendix*, where we analyze effect of drugs on cells as a function of application current and of firing rates).

All of the reported results were unchanged when nonparametric tests (sign rank test and rank sum tests) were employed instead of the parametric *t* test.

We next analyzed how drug application affected cell clusters B1 to B4 and N1 to N3 (*SI Appendix*, Fig. S8). ACh differentially affected the different clusters (*F*(6,63) = 3.72, *P* < 0.001, 1-way ANOVA, *SI Appendix*, Fig. S8). Excitability was not differentially affected by muscarinic (*F*(6,73) = 0.3, *P* = 0.935, 1-way ANOVA) or nicotinic blockade (*F*(6,69) = 1.06, *P* = 0.393, 1-way ANOVA) across the 7 cell clusters. This is likely a consequence of diminished sample sizes that occur with clustering. To follow up on this, we calculated effect size of drug application (*SI Appendix*). Effect size of drug application between clusters often differed more than those between broad and narrow spiking cells, where statistical differences were found upon muscarinic receptor blockade (*SI Appendix*, Table S3).

### Drug Effects on Attentional Modulation of Neuronal Activity.

Attentional modulation emerged after cue onset, and it was most pronounced when the activity was aligned to the time of the first dimming (see Fig. 2 and *SI Appendix*, Fig. S7 for single-cell and population examples). Attention to the receptive field increased firing rates and attention away from the receptive field decreased firing rates relative to the precue activity levels (*SI Appendix*, Fig. S7, cue alignment). However, at the single-cell level some cells decreased their activity during attend RF conditions relative to precue activity and in some cases also relative to attend away conditions, as reported previously (33).

We first describe effects of cholinergic contributions to attentional modulation separately for narrow spiking and broad spiking cells. We quantified attentional modulation by calculating the AUROC for the attend RF vs. attend away conditions, as well as by calculating Cohen’s d′. We included cells where drug application had a significant effect on neuronal activity and where attention had a significant effect on neuronal activity, as we were interested in the drug effect (factor 1) on attentional modulation (factor 2) of firing rates. We focused on the time period before the first dimming, as attentional modulation was strongest for this period, and our interest was to determine how this was affected by the drug of interest.

Given that both factors affected the majority of cells (*SI Appendix*, Table S1), including all cells (even those where 1 of the 2 factors was not significant) yielded qualitatively similar results, with similar significance levels. Attentional AUROC significantly depended on drug application (*F*(3,382) = 11.8, *P* < 0.001, mixed-model [MM-]ANOVA) and cell type (*F*(1,382) = 6.8, *P* = 0.009, MM-ANOVA). Moreover, there was a significant interaction between drug application and cell type (*F*(3,382) = 3.5, *P* = 0.016, MM-ANOVA; the factor “attention” is included in the single dependent variable AUROC and is thus not a separable factor in the ANOVA). To understand how different drugs affected attentional modulation, we separately analyzed attentional modulation given the drug applied for broad and for narrow cells. As stated previously, this was done on the cell sample where attention and drug application significantly affected firing rates.

**Fig. 4.**
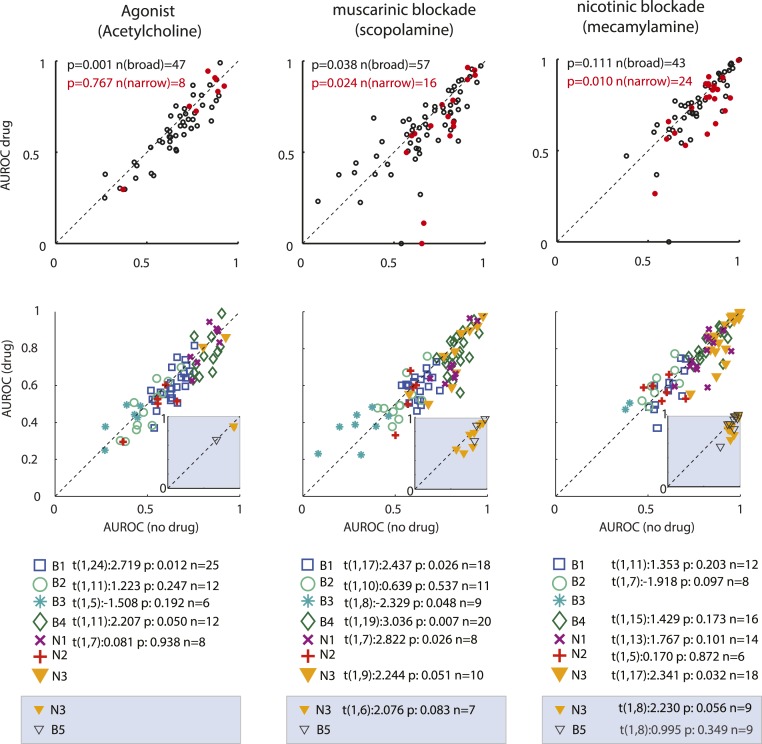
Attentional modulation quantified by calculating the AUROC when no drug (abscissa) was applied and when the drug of interest was applied (ordinate). (*Top* row) Red data points delineate AUROC values of narrow spiking cells and black data points those of broad spiking cells. Significance of effects is given by the *P*-value *Insets*. *n* denotes sample sizes. (*Bottom* rows) Attentional modulation (AUROC) for the different cell clusters in the absence and presence of different drugs. The different cell clusters (B1 to B4 and N1 to N3) are indicated through different colors and symbols (tabulated along with sample sizes and significance of drug effects at the bottom of each subplot). *Insets* in the scatterplots show the further subdivision of the N3 group into cells that were of narrow spiking type and those that were of broad spiking type (B5). Boxes at the bottom of the subplots show associated sample sizes along with *t* statistics (provided *n* ≥ 6) regarding the drug effects on attentional modulation. *n* denotes sample sizes.

Fig. 4*A* shows attentional AUROC values for the 3 drug conditions for broad and narrow spiking cells. Narrow spiking cells showed larger attentional AUROC values than broad spiking cells (*t*(1,193) = −3.574, *P* < 0.001, 2-sided *t* test). ACh application resulted in reduced AUROCs in broad spiking cells (*t*(1,46) = 3.706, *P* < 0.001, 2-sided *t* test). This was because attend away condition activity increased more than attend RF condition activity (*t*(1,46) = −3.217, *P* = 0.002, comparison of drug-induced activity change for attend RF conditions vs. attend away conditions, 2-sided *t* test). No differences were found in narrow spiking cells (*t*(1,7) = 0.308, *P* = 0.767, 2-sided *t* test). Blocking muscarinic receptors reduced attentional AUROCs in broad and narrow spiking cells (*t*(1,56) = 2.127, *P* = 0.038 and *t*(1,15) = 2.510, *P* = 0.024, respectively, 2-sided *t* test, Fig. 4*A*). Blockade of nicotinic receptors reduced attentional AUROCs only in narrow spiking cells (*t*(1,23) = 2.827, *P* = 0.010, 2-sided *t* test), not in broad spiking cells (*t*(1,42) = 1.628, *P* = 0.111, 2-sided *t* test). Thus, muscarinic receptor activation is required for attentional modulation of firing rates in both cell types, while nicotinic receptor activation is required only for attentional modulation of firing rates in narrow spiking, but not in broad spiking cells.

In Fig. 4*A* a minority of cells show AUROC values of <0.5 in the no drug condition. This occurred in cells which showed activity reduction after cue onset, whereby the reduction was larger for attend RF than for attend away conditions. It might be argued that for these cells the AUROC should be calculated as (1 − AUROC). Doing so does not change the conclusions (see *SI Appendix* for associated *P* values).

Similar, but not fully identical, results occurred when we quantified attentional modulation using Cohen’s D. ACh reduced attentional Cohen’s D in broad spiking cells (*t*(1,46) = 2.489, *P* = 0.017, 2-sided *t* test), but not in narrow spiking cells (*t*(1,7) = −0.183, *P* = 0.860, 2-sided *t* test). Muscarinic receptors blockade significantly reduced attentional Cohen’s D in narrow spiking cells (*t*(1,15) = 3.169, *P* = 0.007, 2-sided *t* test), but not in broad spiking cells (*t*(1,56) = 1.384, *P* = 0.172, 2-sided *t* test). Nicotinic receptors blockade significantly reduced attentional Cohen’s D in narrow spiking cells (*t*(1,22) = 2.14, *P* = 0.044, 2-sided *t* test), but not in broad spiking cells (*t*(1,43) = 1.188, *P* = 0.241, 2-sided *t* test).

We next performed this analysis for cell clusters B1 to B4 and N1 to N3. ACh reduced attentional modulation in clusters B1 and B4, but did not affect attentional modulation in the other clusters (see Fig. 4 for visualization and all *t* statistics). Some of these null results may be due to reduced sample sizes caused by clustering. We thus calculated effect sizes to allow for more sample size-independent comparisons (*SI Appendix*, Table S4). Muscarinic blockade significantly reduced attentional modulation in clusters B1, B3, B4, and N1, with trending effects for N3 (Fig. 4). These trending results prevailed when we further subdivided N3 into the narrow (*P* = 0.083, Fig. 4) and broad spiking groups (not tested due to small sample size; *n* = 3). Nicotinic receptors blockade resulted in a significant reduction of attentional modulation in cluster N3, which was predominantly driven by an AUROC reduction in the narrow spiking subgroup of N3 (Fig. 4, *Insets*). Mild trends also occurred for cluster B2 (Fig. 4). However, if anything, nicotinic blockade increased attentional modulation in this subgroup (most data points were above the diagonal).

### Firing Rate Variability as a Function of Attention and Drug Application.

**Fig. 5.**
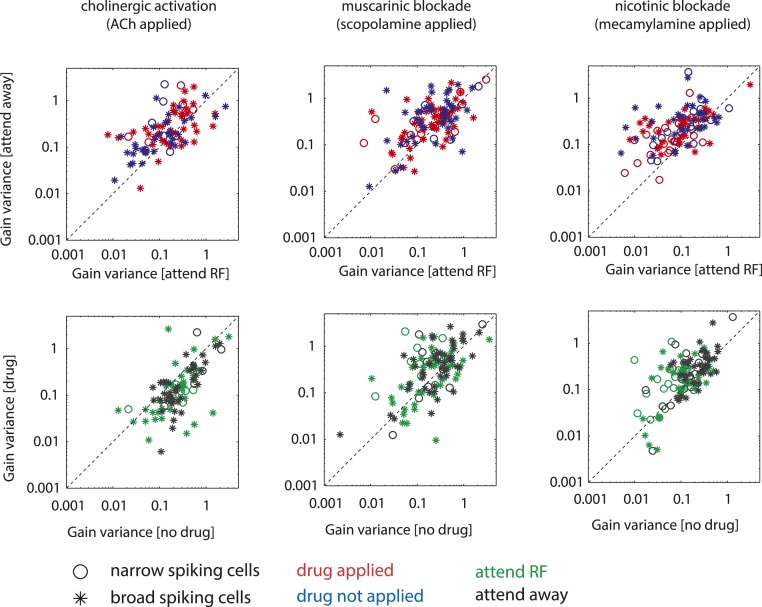
Neuronal variability quantified by gain variance for the different drug and attention conditions. (*Top* row) Effect of attention on gain variance under control conditions (blue) and when the drug of interest was applied (red). (*Bottom* row) Effect of drug application under the 2 attention conditions (green, attention to the RF; black, attention away from the RF). *Left* column shows data for experiments where ACh was applied, *Center* column shows data when scopolamine was applied, and *Right* column shows data when mecamylamine was applied. Circles show data for narrow spiking cells and asterisks data for broad spiking cells.

Attention reduces firing rate variability in addition to affecting firing rates of neurons in visual cortex (7–9). Rate variability was quantified by calculating gain variance (33, 43). For justification of using this measure (and its comparison to Fano factors) see *SI Appendix*. We calculated gain variance as a function of attention and drug application for each cell (Fig. 5). Gain variance significantly depended on cell type (*F*(1,473) = 29.9, *P* < 0.001, mixed-model ANOVA, main effect of cell type). It was lower in narrow spiking cells (*t*(1,213) = −2.326, *P* = 0.021, 2-sided *t* test FDR-corrected post hoc comparison). Location of attention had a significant main effect on gain variance (*F*(1,473) = 20.1, *P* < 0.001, mixed-model ANOVA, main effect of attention, Fig. 5). Attention to the neuron’s RF resulted in lower gain variance (*t*(1,242) = −4.834, *P* < 0.001, 2-sided paired *t* test, FDR-corrected post hoc comparison). Moreover, drug application significantly affected gain variance (*F*(1,473) = 7.0, *P* < 0.001, mixed-model ANOVA, main effect of drug). In addition, there was a trend toward a cell type × drug interaction (*F*(3,473) = 2.3, *P* = 0.08, mixed-model ANOVA), and there was a significant interaction between cell type, attention, and drug (*F*(3,473) = 3.5, *P* = 0.015, mixed-model ANOVA). Other interactions were not significant. The significant interaction might suggest that the effect of a specific drug on gain variance differed between attention conditions. However, this was not the case when analyzed individually for the 3 drugs (cell type × attention × drug interaction: ACh *F*(1,209) = 0.4, *P* = 0.539; Scop *F*(1,197) = 0.3, *P* = 0.589; Mec *F*(1,181) = 0.7, *P* = 0.396). Thus, the effect of the drug on gain variance did not differ between attention conditions in narrow or broad spiking cells (additional details in *SI Appendix*, Fig. S9).

Post hoc testing to determine how the different drugs affected gain variance revealed that ACh did not affect gain variance (see Fig. 5 and *SI Appendix*, Table S6 for exact *P* values, means, and SEM). Scop and Mec significantly increased gain variance in broad and narrow spiking cells (see *SI Appendix*, Fig. S8 and Table S6 for exact *P* values, means, and SEM).

Next, we calculated drug gain variance MIs to determine whether gain variance was more affected by drug application in narrow than in broad spiking cells: $\left(Drug\;Gain\;MI=\frac{Gain\;Var_{no\;drug}-Gain\;Var_{drug}}{Gain\;Var_{no\;drug}+Gain\;Var_{drug}}\right).$ No significant differences were found with ACh applied (*t*(1,70) = −0.504, *P* = 0.616, 2-sided *t* test, broad drug gain MI 0.15 ± 0.06 SEM, narrow drug gain MI 0.23 ± 0.13 SEM). However, gain variance increase was larger in narrow than in broad spiking cells with muscarinic receptors blocked (*t*(1,80), = 2.084, *P* = 0.040, broad drug gain MI −0.08 ± 0.05 SEM, narrow drug gain MI −0.33 ± 0.13 SEM) and with nicotinic receptors blocked (*t*(1,88), = 2.058, *P* = 0.043, broad drug gain MI −0.13 ± 0.05 SEM, narrow drug gain MI −0.30 ± 0.06 SEM). For distributions of drug gain MIs see *SI Appendix*, Fig. S10.

Finally, we determined whether attention or drug application differently affected gain variance in cell clusters B1 to B4 and N1 to N3. Attention MIs of gain variance were determined for control and drug-applied conditions. Attentional MI of gain variance significantly differed between different cell clusters (mixed-model ANOVA, effect of cell cluster: *F*(6,128) = 10.1, *P* < 0.001), but there was no effect of drug application on the attentional modulation of gain variance (effect of drug: *F*(1,128) = 1.8, *P* = 0.122), and there was no interaction (cell type–drug interaction: *F*(12,128) = 0.6, *P* = 0.801). Thus, while drugs affected gain variance, they did not affect the attentional modulation thereof. This differs from their effect on attentional rate modulation (presented above). For additional details see *SI Appendix*, Fig. S11.

### Drug Effects on Behavioral Performance.

Given the local drug application, we did not expect drugs to alter performance. While this proved true for hit and correct rejection rates (*SI Appendix*, Fig. S1), drug application affected reaction times (RTs). We normalized RTs from each session relative to the session mean. This was done as stimulus size and stimulus location varied with RF location and size, which affected RTs between sessions. ACh application showed a trend of reducing RTs (2-factor ANOVA, drug *F*(1,19574) = 3.0, *P* = 0.082, attention *F*(1,19574) = 0.4, *P* = 0.531, drug × attention *F*(1,19574) = 2.4, *P* = 0.123). This trend appeared to be restricted to the attend RF condition (*SI Appendix*, Fig. S12 and Table S7).

Blocking muscarinic receptors caused a significant interaction between the locus of attention and drug application (attention × drug: *F*(1,31641) = 5.0, *P* = 0.025, 2-factor ANOVA), by increasing RTs for the attend RF condition (*t*(1,10546) = 2.157, *P* = 0.031, 2-sided *t* test; *SI Appendix*, Fig. S12 and Table S7), but not for attend away conditions (*t*(1,21091) = −0.857, *P* = 0.315, 2-sided *t* test). Blockade of nicotinic receptors resulted in a trending interaction between the locus of attention and drug application (*F*(1,25980) = 3.3, *P* = 0.069, ANOVA), which differed from the effects seen when Scop was applied. With nicotinic receptors blocked, attend RF RTs were not affected, while attend away RTs showed a mild trend of being increased (*t*(1,17314) = −1.679, *P* = 0.090, 2-sided *t* test; *SI Appendix*, Fig. S12 and Table S7).

## Discussion

Acetylcholine affects the excitability of broad and narrow spiking cells in FEF by activating muscarinic and nicotinic receptors. Muscarinic receptor activation in broad and narrow spiking cells contributes to attentional modulation of firing rates, while nicotinic receptors contribute to attentional modulation only in narrow spiking, but not in broad spiking cells. Activation of both receptor types reduces firing rate variability. The effects were restricted to specific subgroups of narrow and broad spiking cells, which differentiate along the dimensions of attentional modulation, firing rate, and regularity of spiking. These data demonstrate that both receptor types are important in generating attentional signals in FEF, even if muscarinic activation was more critical. The latter is underscored by the finding that blockade of muscarinic receptors increases reaction times for attend RF conditions, which was not found when nicotinic receptors were blocked.

### Cholinergic Contribution to Neuronal Excitability.

In line with previous studies (20, 44–50) ACh increased neuronal excitability. The increased excitability is caused by muscarinic and nicotinic receptor activation in broad and narrow spiking cells. Excitability was more strongly dependent on muscarinic receptors in narrow spiking cells than in broad spiking cells. Does that mean parvalbumin positive (PV) interneurons in FEF are more susceptible to muscarinic activation than other cell types? Almost certainly not. While GABAergic parvalbumin fast spiking interneurons are generally narrow spiking neurons, the opposite cannot be inferred. Narrow spiking cells comprise different cell types (51, 52) and in primates are often even long-range projecting pyramidal cells (36, 53). Thus, a large fraction of the narrow spiking cells in our sample are likely pyramidal cells, narrow spiking calbindin cells, and potentially others. This is corroborated by the cluster analysis, which yielded 3 different narrow spiking cell groups, whereby only 1 cluster exhibited features generally assigned to fast spiking PV interneurons, namely high firing rates and relatively regular interspike intervals (small Lv values). The other 2 cell groups consisting mostly of narrow spiking cells (N1 and N2) show activity characteristics that are rather different and are thus probably different cell types (possibly narrow spiking pyramidal cells and narrow spiking calbindin/calretinin cells). However, cluster N3, which exhibited features often assigned to PV interneurons, consisted of different cells with respect to the spike waveform width. One subgroup exhibited very narrow spikes. The other subgroup had slightly wider spikes, which usually fall into the broad spiking category. It is tempting to speculate that the narrow subgroup from cluster N3 mostly consists of PV fast spiking interneurons. This would match the number of PV cells expected to occur in an opportunistic sample of cell recordings. Twenty-five percent of cortical neurons are inhibitory cells (40), of which ∼25% are PV neurons (40); an opportunistic sampling would yield 6.25% PV cells in randomly recorded cells from the cortex. The narrow spiking cells from the N3 group constitute 6.9% of all recorded cells, which is in good agreement with this expectation. However, microelectrodes have a sampling bias for larger cells/action potentials and we would expect to undersample PV cells, with even fewer PV cells than narrow spiking cells present in the N3 cluster. Given these considerations, it is impossible to prove that the N3 sample indeed consists of PV interneurons.

It is even more difficult to assign morphologically or immunohistochemically identified cell types to other clusters and thus to specific cholinergic modulation. The majority of GABAergic interneurons express muscarinic receptors in V1 of the primate, while only few excitatory (pyramidal) cells show muscarinic expression (26). In rodents, muscarinic receptors are more strongly expressed on somatostatin and VIP interneurons than on PV interneurons (54). Since the former 2 types are generally broad spiking, one might expect broad spiking cell excitability to be more affected by muscarinic blockade than narrow spiking cell excitability. Conversely, we found that cell excitability in narrow spiking cells was more strongly affected by muscarinic blockade than broad spiking cell excitability. This is not necessarily surprising for 2 reasons. First, the expression level of muscarinic receptors in excitatory cells increases with cortical hierarchy of the primate, at least when considering V2, area MT, and dorsolateral prefrontal cortex (DLPFC) (26, 28, 55, 56), and thus differences are expected for FEF as well. Second, substantial differences exist in cell type-specific expression of cholinergic receptors between primates and rodents (30). Thus, if a large proportion of pyramidal cells express muscarinic receptors in the FEF, and many of these were narrow spiking (36, 53), our result is not necessarily surprising anymore. However, these scenarios remain speculative.

Without the use of more specific muscarinic agonists and antagonists it is currently impossible to argue which muscarinic receptor subtype might be responsible for our findings. In neighboring DLPFC (area 46), muscarinic M1 and M2 receptors are expressed postsynaptically on symmetric and less often on asymmetric synapses on excitatory cells, but also occur on interneurons (55, 56). Based on figures 1–3 of ref. 56, similar results appear to be the case for M2 receptor expression in FEF. M1 activation results in increased layer V pyramidal cell excitability in prefrontal and sensory cortex of rodents (57, 58). Moreover, activation of M2 receptors results in reduced inhibitory postsynaptic currents, which can have a net effect on increased cell excitability across the population due to the reduction in inhibitory efficacy (58). The reduced excitability of FEF cells in our sample upon muscarinic blockade could thus have been caused by either receptor subtype. Further studies with more specific antagonists and agonists are required to delineate the individual receptor contributions, ideally in combination with cell type-specific labeling techniques (e.g., photo tagging of specific cell types).

### Cholinergic Contribution to Attentional Rate Modulation.

In FEF ACh contributes to attentional signals through muscarinic receptors in broad and narrow spiking cells, while nicotinic receptors contribute to attentional signals only in narrow spiking cells. ACh contributes to attentional rate modulation through muscarinic receptors in area V1, while nicotinic receptors do not (20). Ref. 20 does not differentiate between broad and narrow spiking cells. In principle, nicotinic receptors might also exclusively contribute to attentional modulation in narrow spiking cells in area V1, given that fewer cells are of the narrow spiking type. Mixing a relatively small number of affected narrow spiking cells with a comparatively large number of nonaffected broad spiking cells might eliminate the significance of any changes. However, nicotinic blockade in the Herrero et al. (20) dataset did not result in even a hint of attention-induced rate change reduction (figure 4 in ref. 20), suggesting there may be genuine differences between area V1 and FEF.

The result that nicotinic receptors do not contribute to attentional modulation in FEF broad spiking cells might seem somewhat surprising, given the involvement of nicotinic (α7) receptors in spatial working memory delay activity, through interaction with NMDA receptors on layer 3 DLPFC pyramidal cells (59). But this surprise is under the premise that pyramidal cells in FEF are of broad spiking type and based on the premise that spatial working memory and spatial top–down attention are related concepts (which we believe is the case). Analyzing attentional modulation by nicotinic blockade for the different cell clusters showed that none of the different broad spiking cells alter their attentional modulation when nicotinic receptors were blocked, but pyramidal cells in frontal cortex can be of narrow spiking type (53). Changes of attentional modulation occurred only in the N3 cluster, which comprised a broad and a narrow spiking subgroup. The narrow spiking subgroup of N3 exhibits features compatible with fast spiking PV interneurons. However, these features have been studied in slices from nonprimates. It remains to be determined whether the differences in spike width between primate motor/premotor pyramidal cells and equivalent (rat) cells (36, 53) map onto other activity characteristics as well. Caution is needed to argue that the nicotinic contribution to attentional modulation is mediated through narrow spiking PV interneurons, as we would expect this to “spill over” to pyramidal cell types due to their inhibitory effect on neighboring pyramidal cells. Thus, if our drug manipulations affect nearby PV cells, this effect should also be seen in pyramidal cells, due to the strong coupling of PV cell activity to pyramidal cell activity.

The relative contribution of muscarinic and nicotinic receptors to attentional rate modulation in FEF cells is matched by behavioral studies in humans, where ACh contributes to attention predominantly through muscarinic receptor activation. However, synergistic effects between nicotinic and muscarinic receptor activation were also found (60). Other studies have demonstrated a direct role of α7 nicotinic receptors in sustained attention in mice (61). Our study points to a more profound role of muscarinic receptors in FEF in attentional control also at the behavioral level. Muscarinic blockade affected behavior in our animals, increasing reaction time, while nicotinic blockade had no effect. We currently do not know whether M1 or M2 muscarinic receptors are responsible for these effects. M1 receptors are not responsible for maintenance of working memory signals in a rule-based saccade task in DLPFC (62). The effects seen in our study might thus be mediated by M2 receptors, but it remains to be seen whether the same dependencies apply to a spatial attention task in FEF. We did not employ combined blockade of nicotinic and muscarinic receptors, but the results by Ellis et al. (60) suggest that combined blockade might result in stronger behavioral effects.

Rate variability, as measured by gain variance (43), in broad and in narrow spiking cells was reduced by attention, as previously reported for FEF (33) and area V4 (63). Application of ACh reduced gain variance in broad and narrow spiking cells. Blockade of muscarinic and of nicotinic receptors increased gain variance. Theoretical work suggests that neural variability in neuronal attractor networks during spontaneous activity is high because the attractor networks operate close to criticality, a point where the network is close to a boundary where input noise induces large fluctuations between multiple—unstable—network states (64). Stimulus presentation and directed attention increase excitatory drive and move neurons away from the point of instability (the bifurcation point), thereby stabilizing 1 specific attractor, reducing transitions between different attractor states. This in turn results in a net decrease in neural variability. Our data show that cholinergic activation of muscarinic or nicotinic receptors decreases neuronal excitability, and conversely their blockade results in increased neuronal variability. The excitation induced by stimulus presentation and/or attention is less effective when cholinergic receptors were blocked, resulting in less stable attractor states. Changes in attractor and brain states depend on neuromodulator tone (3, 49, 65–69). We argue that ACh supports attentional states by increasing desynchronized brain states (70). This favors stable attractor configurations (e.g., directions of attention) which are less prone to external perturbation (distractions). In mouse visual cortex these state changes are critically dependent on somatostatin-positive interneurons (71). Whether similar mechanisms are at work in FEF is unknown.

### Comparison to Other Transmitters/Neuromodulators.

While muscarinic and nicotinic receptor blockade increased rate variability in FEF, it did not affect the attentional modulation thereof. However, blockade of either receptor altered attention-induced rate changes. In area V1 attention-induced alterations of rate variability are dependent on NMDA receptor activation (8). Whether NMDA receptor activation has similar effects in area FEF is currently unknown. Drug infusion studies into FEF suggest that dopamine affects behavior and activity in area V4 by acting on FEF D1 receptors, causing effects similar to those of attention (72). This occurs by affecting the strength of inputs to and recurrent connectivity within FEF (73). In DLPFC dopamine, NMDA, α7 nicotinic receptors, and noradrenergic α2a receptors play important roles in the generation of spatial working memory signals (59, 74, 75). These are conceptually related to covert spatial attention signals. By acting on D1 receptors dopamine supports working memory-related activity through noise reduction (76), while noradrenergic α2a receptor activation enhances working memory-related activity through signal enhancement (75). NMDA and α7 nicotinic receptor activation are required to generate persistent delay activity in the absence of sensory stimulation (59, 74). Our data suggest that cholinergic stimulation in FEF has a role similar to α2a and NMDA receptor activation, supporting a persistently enhanced activity of the attended stimulus. Together these studies suggest that it is the combined action of multiple transmitter/neuromodulator systems that shapes the frontal network activity, thereby supporting cognitive functions. In relation to cholinergic influences, our study shows that attentional signal generation in FEF is more reliant on muscarinic receptors and less on nicotinic receptors. This difference may have implications for future treatment strategies of attentional dysfunction.

## Methods

### Experimental Procedures.

Procedures were approved by the Newcastle University Animal Welfare Ethical Review Board (AWERB) and carried out in accordance with the European Communities Council Directive RL 2010/63/EC, the US National Institutes of Health Guidelines for the Care and Use of Animals for Experimental Procedures, and the UK Animals Scientific Procedures Act.

#### Surgical preparation.

Monkeys were implanted with a headpost and recording chambers over area FEF under sterile conditions and under general anesthesia, as published in detail previously (77).

#### Identification of recording sites.

Area FEF was identified by means of structural MRI. FEF recording sites were confirmed by visual RF size and topography (78), by memory-guided saccade responses, by saccade-related responses to the visual/motor field, and by means of low-current (50 µA) electrical saccade induction (78). Additionally recording sites in area FEF were verified in histological sections stained for cyto- and myeloarchitecture (79).

#### RF and saccade field mapping.

The location and size of the RF were measured by a reverse correlation method (80). Saccade fields (SFs) were mapped as described in ref. 33.

#### Behavioral task and stimuli.

All details about the task and stimuli have been published previously; for ease of access they are repeated in *SI Appendix*.

### Electrophysiological Recordings and Drug Application.

Drugs were applied iontophoretically using a tungsten-in-glass electrode flanked by 2 pipettes (77). For details regarding electrodes, cell inclusion, and number of trials available under different drug conditions see *SI Appendix*.

### Data Collection.

Stimulus presentation and behavioral control were managed by Remote Cortex 5.95 (Laboratory of Neuropsychology, National Institute for Mental Health, Bethesda, MD; http://dally.nimh.nih.gov/). Neuronal data were collected by Cheetah data acquisition (Neuralynx) interlinked with Remote Cortex. For details regarding data acquisition and postprocessing see *SI Appendix*.

### Data Analysis.

We analyzed neuronal activity associated with correct trials. We aligned neuronal activity to the stimulus, to the cue, and to the first dimming onset. To analyze the effects of attention on neuronal firing rate, we analyzed the activity from −500 ms to 0 ms before the first dimming. Given that there were 3 attention conditions (attend RF and 2 attend away conditions), 2 different stimulus motion directions, and 2 drug conditions (applied vs. not applied) we had 6 conditions total for each drug condition. We calculated a 3-factor ANOVA for the predimming activity to determine whether attention, drug application, and direction of motion had a significant effect on neuronal activity and whether there was a significant interaction between any of these factors. Cells with a significant main effect of attention or a significant interaction (*P* < 0.05) were classified as attention modulated; cells with a significant main effect of drug application or an interaction of drug applications with any of the other factors were classified as drug modulated.

### Quantification of Attentional Modulation.

The strength of attentional modulation was quantified using an ideal observer-based approach, whereby we calculated the AUROC separately for the no drug condition and the drug condition. It is based on signal detection theory which calculates the overall probability that a random sample of neuronal activity (i.e., spikes per second) selected during one attention condition is larger than a sample selected in the alternative attention condition (81–83).

### Quantification of Drug Effects.

Drug effects were assessed by calculating a drug MI for each stimulus condition and for each analysis period (100 to 400 ms after stimulus onset, 100 to 400 ms after cue onset, and −500 to 0 before the first dimming):

$$Drug\;MI=\frac{Activity\;no\;drug\;-Activity\;drug}{Activity\;no\;drug+Activity\;drug}.$$

### Gain Variance Analysis.

To investigate to what extent the different drugs affected attention-induced changes in response reliability, we used both Fano factor and gain variance analysis (43). In gain variance analysis, the single-trial rate (count data) is fitted with a negative binomial, which yields a gain variance term. This captures the magnitude of the change in excitability from trial to trial. We used the 2 attend RF conditions to obtain an estimate of the attend RF gain variance and the 4 attend away conditions to obtain an attend away gain variance estimate. Gain variance terms were then averaged for the 2 attend RF conditions and separately for the 4 attend away conditions. This was done separately for the drug applied and drug not applied conditions.

### Cluster Analysis.

We followed the procedures published by Ardid et al. (42) and used the open source Matlab resources (https://bitbucket.org/sardid/clusteringanalysis) with minor modifications to suit our data. For all details regarding the cluster screening, the parameters used for final clustering, and the clustering itself see *SI Appendix*, *Methods*.

### Analysis of Behavioral Data.

We calculated control condition and drug condition reaction times and error rates for each experimental session where a significant effect of drug application was found at the cellular level. For details see *SI Appendix*.

## Supplementary Material

Supplementary File
