# Supplementary material for: Cell class-specific modulation of attentional signals by acetylcholine in macaque frontal eye field
Source: Proc Natl Acad Sci U S A. 2019 Sep 16;116(40):20180–9. doi: 10.1073/pnas.1905413116 (PMC6778228; doi:10.1073/pnas.1905413116)
Supplement: Supplementary File [file pnas.1905413116.sapp.pdf]

Supporting Information (SI) Appendix:

Cell class specific modulation of attentional signals by acetylcholine in macaque frontal eye field

Miguel Dasilva[1,4], Christian Brandt[1,3], Sascha Gotthardt[1], Marc Alwin Gieselmann[1], Claudia Distler[2], Alexander Thiele[1]*

[1]Institute of Neuroscience, Newcastle University, UK

[2] Allgemeine Zoologie und Neurobiologie, Ruhr-Universität Bochum, 44801 Germany

[3] current address: Institute of Clinical Research, University of Southern Denmark, DK

[4] current address: College of Medicine and Health, University of Exeter, UK

* Corresponding and senior author: Alexander Thiele, Institute of Neuroscience, Newcastle University, Newcastle upon Tyne, NE2 4HH. E-mail: alex.thiele@ncl.ac.uk, Orcid ID: https://orcid.org/0000-0003-4894-0213

www.pnas.org/cgi/doi/10.1073/pnas.1905413116

SI Appendix Methods

Animals

We used two adult awake male macaques (*Macaca mulatta*, age 5-9 years, weight 11-15 kg). Animals were motivated to engage in the task through fluid control at levels that do not affect animal physiology and have minimal impact on psychological wellbeing [1].

Behavioural Task and Stimuli

Monkeys fixated a white fixation point on a grey background presented centrally on a 20" analogue cathode ray tube monitor (110 Hz, 1,600 × 1,200 pixels, 57 cm from the animal). Eye position was monitored with an infrared based system (Thomas Recording, 220 Hz) with a fixation window of ±0.7° - 1.5°. The monkey initiated trials by holding a touch bar and fixating on the central point (figure 1 main manuscript). 500 ms after fixation onset 3 stimuli appeared on the screen, equidistant from the fixation spot. One stimulus was centered on the RF of the recorded neuron. The other stimuli were presented equidistant on an invisible circle centered on the fixation spot. Stimuli were square wave gratings (1 cycle/deg duty cycle) in a circular aperture sized according to the RF size. One grating was red/grey, one green/grey and one blue/grey. Locations of differently coloured gratings were fixed within a recording session, but differed pseudo-randomly between recording sessions. Grating orientation was fixed within a session, but differed randomly between sessions. Gratings moved perpendicular to the orientation (1 Hz temporal frequency). The motion direction (perpendicular to the orientation) was pseudo-randomly assigned on each trial. After a randomly selected time of 300-1400 ms a central cue of either green, blue or red colour appeared. The cue colour indicated which of the 3 gratings would be behaviourally relevant on the current trial (the colour matched grating). Cue selection occurred pseudo-randomly. After 600-1750 ms one pseudo-randomly selected grating was reduced in luminance (for details see [2]). If the cued grating had changed luminance, the monkey had to release a central touch bar within 600 ms to obtain a fluid reward. If an un-cued grating had changed luminance, the animal had to ignore it and wait for the cued grating to change luminance. This could happen after 600-750 ms after the first dimming, or 600-750 ms after the second dimming (figure 1a, main manuscript). Throughout the entire period, the monkey had to fixate on the central fixation spot. The task had no catch trials, i.e. the cued grating always changed luminance, but the order thereof was unpredictable up to the point when the second grating had changed luminance. The timing of the dimming was also unpredictable, within the time period indicated above.

Data collection and post processing

Raw data were acquired at a sampling frequency of 32556 Hz with a 24-bit analog-to-digital converter, with minimum and maximum input ranges of 11 and 136986 microvolts respectively (pre-set by Neuralynx, Inc.), a DMA buffer count of 128, and a DMA buffer size of 10 ms, using a 64-channel Digital Lynx 16SX Data Acquisition System (Neuralynx, Inc.). Digital referencing of voltage signals was performed prior to the recording of raw data, using commercially-provided Cheetah 5 Data Acquisition Software v. 5.4.0 (Neuralynx, Inc.).

Following each recording session, the raw data were processed offline using both commercial (Neuralynx, Inc.) and custom-written (Matlab, Mathworks) software. Signals were extracted using Cheetah 5 Data Acquisition Software. The sampling frequency remained the same (32556 Hz), while the input range settings were individually tailored to session, with band pass filter frequency set to a low cut frequency of 600 Hz and a high cut frequency of 9000 Hz, and saved at 16-bit resolution.

Electrophysiological recordings and drug application

Drugs were applied iontophoretically using a tungsten-in-glass electrode flanked by two pipettes [3]. The tungsten in glass electrode had impedances of 0.5-1.5MΩ (measured at 1 kHz), and an exposed tungsten tip of <10µm. Pipette opening diameter varied between 1-4 µm.

Neurons were further analysed if at least 10 trials per condition were available. For the vast majority of recordings we obtained >30 trials per attention and drug condition (after removal of 'transition trials'). The median number of trials for our ACh recordings were n=51 per condition (25, 75%ile: n=36, n=63). For the scopolamine recordings the median number of trials was n=47 per condition (25, 75%ile: n=32, n=59). For the mecamylamine recordings the median number of trials was n=54 per condition (25, 75%ile: n=35, n=61).

Pipette resistance varied between 10-400 MΩ (median: 35 MΩ, 25-75%ile: 26-50 MΩ). The integrity of the electrode and the pipettes were checked under the microscope before and after the recording sessions, in addition to measurements of the pipette impedance made before and after the recording at each recording site. The details regarding drug concentration, pH and application current were: Hold currents for acetylcholine (0.1M, pH 4.5) were -32nA (median, 25-75%ile: -40 to -15 nA; minimum: -50nA; maximum: -5nA), ejection currents were usually 20nA (median; 25-75%ile: 10 to 30nA; minimum: 1nA; maximum: 60nA). Hold currents for scopolamine (0.1M, pH 4.5) were -15nA (median, 25-75%ile: -20 to -10 nA; minimum: -50nA; maximum: -5nA), ejection currents were usually 20nA (median: 20nA; 25-75%ile: 15 to 40nA; minimum: 1nA; maximum: 80nA). Hold currents for mecamylamine (0.1M, pH 4.5) were -

11nA (median, 25-75%ile: --15 to -8 nA; minimum: -47nA; maximum: -1nA), ejection currents were usually 15nA (median: 20nA; 25-75%ile: 12 to 20nA; minimum: 5nA; maximum: 60nA).

Drug application was continuous during blocks of ‘drug applied’. Each block lasted for at least 36 trials (2 repetitions of: 3 attention locations* 2 directions of motion * 3 dimming times=36), with error trials repeated at random times within a block, such that block length depended to some extent on monkey performance. On average, drug/no drug application for each block was ~3-9 minutes. For the data analysis, we removed the first 6 trials within a block from the data set, as drug effects and recovery usually occur with a slight delay of ~0.5 minutes. In control experiments (n=17) we used saline in the pipettes (0.9%, pH 4.5), using similar hold and ejection currents (-20nA hold, 15 nA eject) and determined whether it affected neuronal activity. In these control experiments, we did not see significant effects of saline application on firing rate or attentional modulation.

The fact that currents for the three drugs and the pH were identical during hold and application conditions, while the effects on neuronal activity and attentional modulation differed significantly between the three drugs, further argues against the possibility that application current or pH could have contributed to our findings.

SI Appendix Extended data

Animal performance under control and drug conditions

We calculated control condition and drug condition reaction times and error rates for each experimental session where a significant effect of drug application was found at the cellular level. This selection was performed, as the method of drug application does not guarantee that drugs are adequately infused in every experimental session. The selection ensures that only sessions are included, where we have an independent verification that the drugs affected the neuronal tissue, a prerequisite to cause changes at the behavioural level, without pre-empting that a behavioural effect did occur, or its direction.

Error numbers were calculated separately for the different attention conditions and for the different drug conditions. All fixation errors were discarded, i.e. we only included trials where the animal reported a distracter dimming, instead of a target dimming, or where the animal did not report a dimming at all (miss). From these data we calculated the hit rate and correction rate as described below.

The animals' performance was assessed by calculating hit rates (p(hit)) and correct rejection (p(CR)) rates for each experimental session according to:

$$p(hit) = \frac{number\ of\ hits}{number\ of\ hits + number\ of\ omissions}$$

$$p(CR) = \frac{number\ of\ CR}{number\ of\ CR + number\ of\ false\ alarms}$$

hits were defined as correct target dimming reports, omissions were defined as missed target dimmings. Correct rejections (CR) were defined as unreported distracter dimmings, while false alarms were defined as reported distracter dimmings.

These were calculated separately for each of the three stimulus locations, and separately for the control and the drug applied conditions. Overall hit rate and correct rejection rates were very high (figure S1), indicating that the animals had learned the task and heeded the attention cue. Single session hit rates and correct rejection rates were used to calculate whether drug application affected animal performance (mixed model ANOVA with attention and drug application as factors).

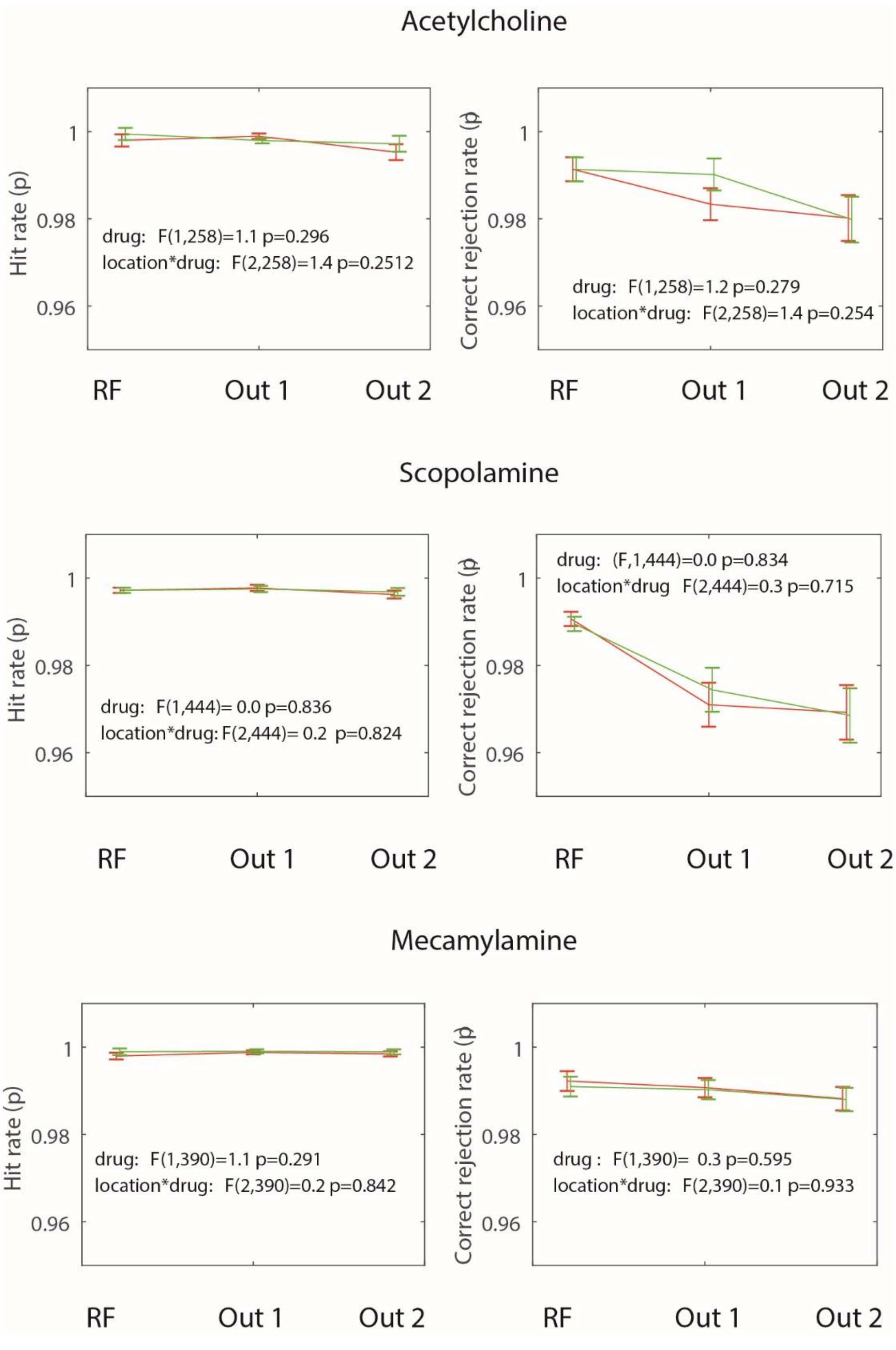

Figure S1: Animal performance across the three potential dimming locations for control (red) and drug applied conditions (green). Hit rates (p(hit)/(p(hit)+p(miss)) and correct rejection rates (p(correct rejection)/(p(correct rejection)+p(false alarm)) were calculated for each recording session separately. Error bars show means +/- S.E.M. across experimental session. RF indicates that the dimmings happened at the receptive field location. Out1 and Out 2 indicate that they happened at one of the other 2 possible locations.

Analysis of cell type along the broad narrow spiking divide

To classify cells as broad or narrow spiking, we performed spline interpolation of the original waveforms to obtain a resolution of 5.4 µs [4]. We used peak to trough time as a classification criterion. In our sample a cut off of 250 µs peak to trough times was appropriate, as this cut off separated the significantly bimodal distribution (calibrated Hartigan's dip test $p<0.001$) of peak to trough times, while at the same time reducing the risk of classifying narrow spiking cells as broad, since the cut off was located to the narrow spiking side of the bimodal separation (see [2] for details).

Number of cells recorded from the 2 monkeys under the different drug regimes

	Cell type (spike)	Drug type	Total n	Attention effect (n)	Drug effect (n)	Attention & drug effect (n)
Monkey 1	narrow	ACh	8	5	6	4
	broad	ACh	30	23	23	18
	narrow	Scopolamine	21	18	12	10
	broad	Scopolamine	27	22	14	11
	narrow	Mecamylamine	28	26	19	19
	broad	Mecamylamine	27	22	17	14
Monkey 2	narrow	ACh	5	5	5	5
	broad	ACh	43	32	36	29
	narrow	Scopolamine	10	8	8	7
	broad	Scopolamine	78	68	51	46
	narrow	Mecamylamine	14	11	9	6
	broad	Mecamylamine	53	45	35	29
Both Monkeys	narrow	ACh	13	10	11	9
	broad	ACh	73	55	59	47
	narrow	Scopolamine	31	26	20	17
	broad	Scopolamine	105	90	65	57
	narrow	Mecamylamine	42	37	28	25
	broad	Mecamylamine	80	67	52	43

SI Appendix Table S1: Number of cells recorded from the 2 monkeys under the different drug regimes, and number of cells that showed significant attention effects, number of cells with significant drug effects, and number of cells that showed significant attention and significant drug effects.

Cluster-analysis for the identification of cell classes in FEF

Identifying redundant and uninformative measures

We followed the procedures established by Ardid et al [5], with the exception of using attentional modulation (quantified by calculating the area under the receiver operating characteristic [attROC]) as an additional clustering variable.

For the clustering we used attentional modulation (quantified by AUROC, see above), spike width, firing rate during the last 500ms before the first dimming in the attend RF condition, and

the regularity of spiking as cluster features. Regularity of spiking was assessed during the last 500ms before the first dimming in the attend RF condition, by assessing the coefficient of variation (Cv) of interspike intervals (ISI). This is calculated as the standard deviation of ISIs, divided by the mean ISI. Very regular ISIs would result in CVs<1, however, locally regular spiking might be obscured if the firing rate was non-stationary, i.e. ISIs gradually change over time. To account for this two additional measures have been introduced, which measure similarity between neighbouring ISIs. The coefficient of variation of neighbouring ISIs is referred to as CV2, and it measures twice the difference between the ISIs, normalized by their sum [6], while the local variation (Lv) measures the squared difference between neighbouring ISIs, normalized by their sum [7].

Clustering was done based on standardized feature values, i.e. all values were normalized to range from 0 to 1, by first subtracting the minimum value from all values and then dividing by the maximum value found in the population. Standardization ensures that all cluster variables have an identical range, and as such have the same basic weight in the process of selecting the appropriate clusters and in the clustering itself. For example, the peak to trough times of spike waveforms range from 102us to 772us across the sample. For standardization we subtract 102 from all values, and then divide by the maximum (which now is 670us after the initial subtraction). Thus the peak to trough time 102us gets assigned a value of 0, and the peak to trough time 772us gets assigned a value of 1, and all other values are in between. Standardization was done separately for each cluster variable.

To minimize the contribution of redundant and uninformative measures to the classification, we calculated the dissimilarity between measures, and we computed how much the different measures contribute to the variance in our data. To reduce possible bias in the clustering we decided to use a cut-off of 0.1 for the dissimilarity (i.e. a Spearman correlation >90% between measures). None of our variables showed correlations of the size, so it was effectively not necessary to exclude any measures based on this criterion (figure S2A). The relative contribution of different measures to the variance in the data is shown in figure S2B. Here we eliminated measures that together represented less than 10% of the total variance. This applied to the measures of Coefficient of variation (Cv) and rate variability as measured using the Fano Factor (FF), leaving attROC, spike width (peak to trough duration, P2T), firing rate (FR), local variability of the interspike interval (Lv), and Coefficient of variation of neighbouring interspike intervals (CV2), as variables used for the clustering of our cell sample.

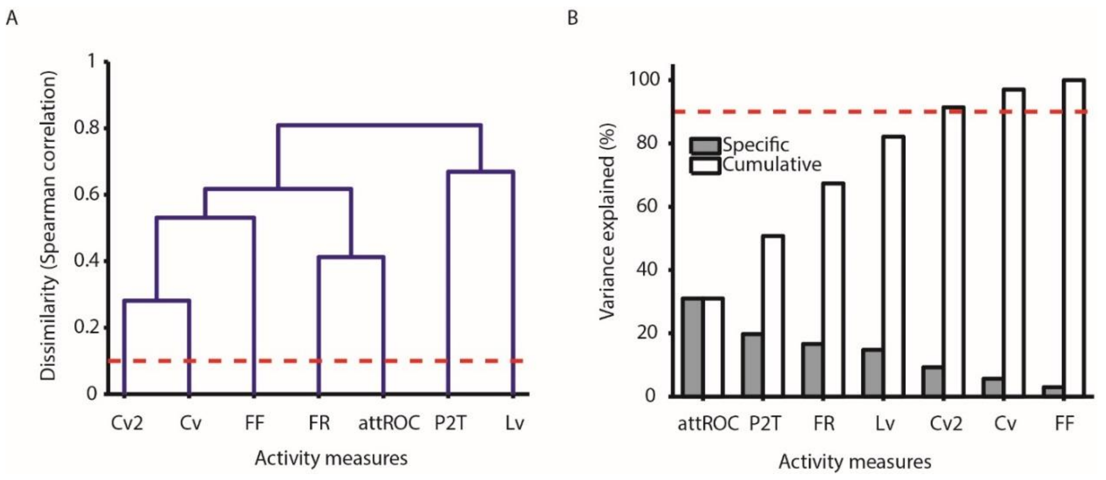

Figure S2: Analysis to determine the degree of correlation between cluster variables (or their dissimilarity), and how informative measures are for clustering. A) Dissimilarity measured using Spearman's correlation. Measures with a level of dissimilarity <0.1 would have been filtered out, due their high level of correlation, which would otherwise have introduced bias. The dendrogram of dissimilarity shows that this was not the case for any of the measures used. B) Variance explained by the different measures (grey bars) and cumulative variance (white bars) explained by the different cell features (measures). Features were ordered according to the amount of variance they explained, and a cut-off of >90% for cumulative variance was chosen to eliminate uninformative features from the final clustering. This approach resulted in elimination of the coefficient of variation of the interspike interval (Cv) and of the Fano Factor (FF).

Identifying the number of cell classes in FEF

We used K-means clustering to identify how many cell classes can be identified based on the physiological features of attROC, P2T, FR, Lv, and CV2. To perform initial identification of the number of clusters to analyse we used 50 replicates of K-means clustering for cluster numbers ranging from 1 to 40. We used the best replicate for each k ('minimum squared Euclidian distance from all cluster elements to their respective cluster centroids' [5]). These procedures were done exactly as described in [5], and the outcome was also very similar to that published for a different set of prefrontal neurons [5], namely an estimate of 5-15 clusters being appropriate for the present dataset (figure S3A).

To select the most appropriate number of clusters from this range of 5-15 we re-run the K-means clustering with n=500 realizations (with 50 replicas for each k and n, selecting the best replicate) each for cluster numbers k=5 to k=15. Probability thresholding that different pairs of neurons belong to the same cluster was done as described in [5], thereby separating reliable from spurious clusters. We only considered clusters if they contained at least 5 cells. Using Akaike (AIC) and Bayesian information criterion (BIC) showed that 7 (AIC) and 6 (BIC) clusters were the most appropriate choice for our data set (AIC values k=5-11: -5623.1, -5642.8, **-5649.6,** -5609.7, -5476.6, -5636.1, -5418.1; BIC values k=5-11: -5414.5, **-5432.9,** -5383.2, -5306.0, -5175.4, -5294.7, -5042.8).

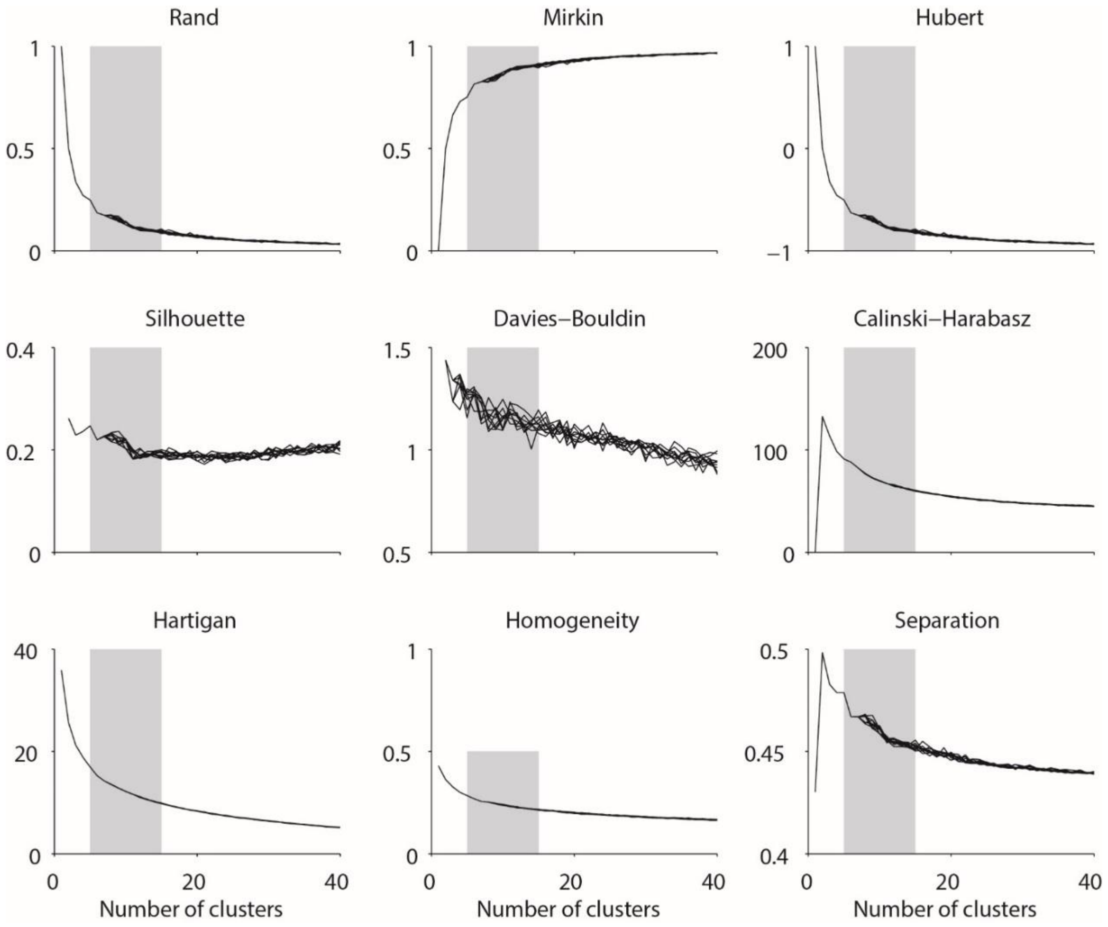

Figure S3: Initial attempt to determine how many clusters can best be detected amongst our cell sample. We estimated the quality of clustering (given a set cluster number [k=1-40]) using the indices named above each subplot. Near asymptotic behaviour occurs for all indices for the range of k=5-15 (shaded area). The different lines in each subplot correspond to the different initializations (n=10).

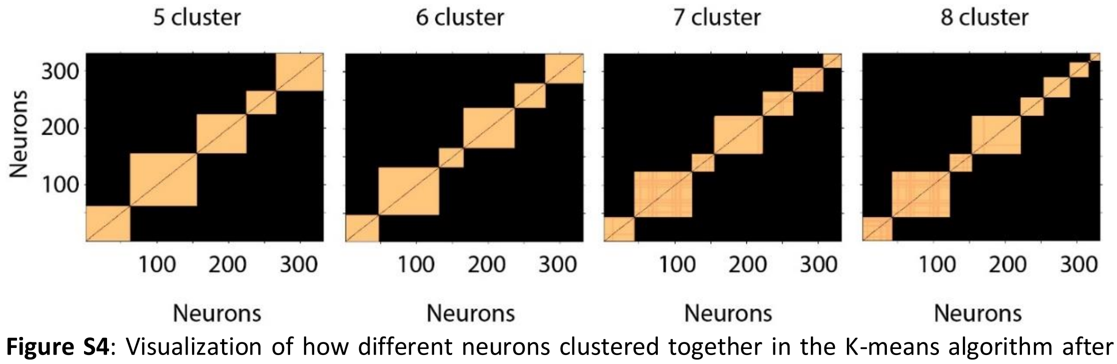

Figure S4: Visualization of how different neurons clustered together in the K-means algorithm after imposing clusters of k=[5,6,7,8]. Details regarding the visualization and clustering of cells are given in [5] (their figure 4B). When k=5-7 no cells remained un-clustered (all clusters contained >5 cells). When k=8, four cells remained un-clustered.

Deciding on the number of clusters given the discrepancy between AIC and BIC measures

As stated above, AIC and BIC differed in their assessment as to the appropriate number of clusters to be applied. While AIC argued for 7 clusters, BIC argued for 6 clusters. The dendrogram based on 7 different clusters along with P2T distributions are shown in figure S5. Using 7 clusters resulted in 4 clusters of broad spiking cells which did not include narrow spiking cells (with a notable exception of n=1). This was based on a narrow-broad spiking divide of P2T of 250 µs. Conversely, narrow spiking clusters did invariably contain some broad spiking cells, whereby the proportion was largest in cluster N3. It is worth noting, that this type of blurring was also present in the prefrontal data set published by Ardid et al. [5] (see their figure 5B), although the lack of absolute narrow-broad boundaries across all cluster was never discussed it in the paper. Imposing 6 clusters (as suggested by the BIC values) resulted in clusters where the broad-narrow spiking divide was even more blurred (figure S5).

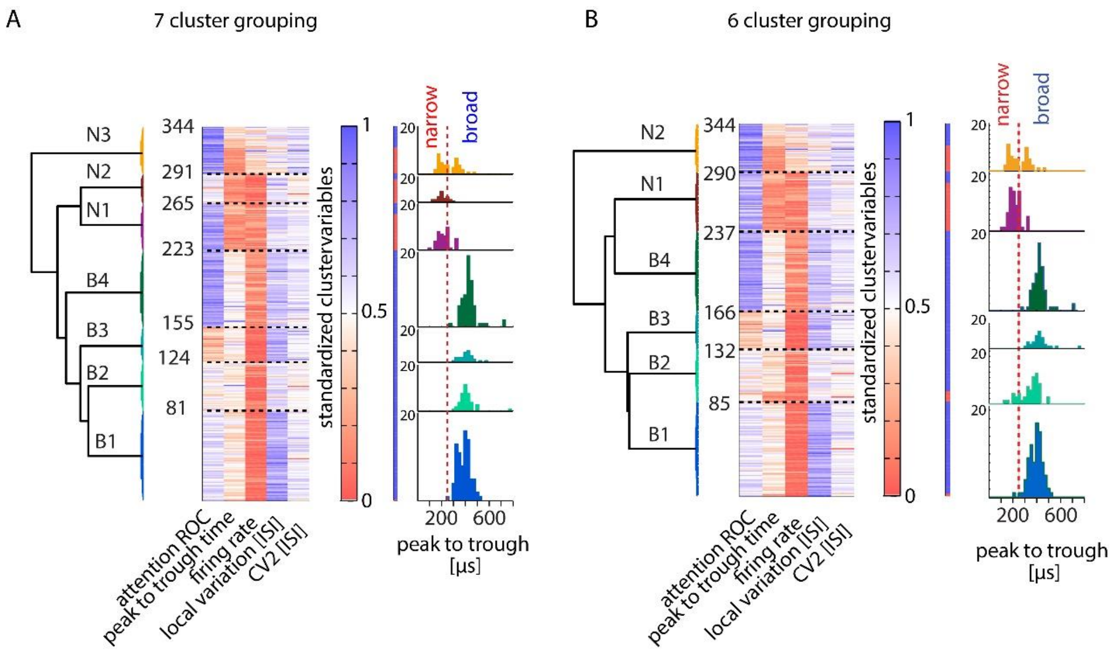

Figure S5: Comparison of grouping after imposing 7 clusters and 6 clusters, respectively. A) Dendrogram on the left shows the linkage between different cell clusters. Heat maps (next to the dendrograms) show the values of different neuronal features used for classification (x-axis) for all clustered cells (y-axis). Color coding is according to increasing standardized feature values (cluster-variables). Dashed lines within heat maps show cell class borders along with cumulative cell numbers (i.e. cluster sizes can be inferred from these numbers). The color bar to the right of the standardized cluster-variable bar, shows encoding of cell type along the narrow-broad spiking divide (narrow: red, broad: blue). Distribution of peak to trough times for each cell cluster are shown on the right of each subplot (scaled to frequency n=20 for each subplot). Red dashed line shows broad-narrow divide used in the current paper (250µs). Clusters B1-B4 mostly comprised of broad spiking cells. Clusters N1-N3 comprised more of narrow spiking cells, however, N3 was significantly bimodal and comprised of broad and narrow spiking cells. B) Same as A, but with 6 instead of 7 clusters.

Cluster features and their association with different cell classes in FEF

As stated above, the physiological cell features used for clustering were attROC, P2T, FR, Lv, and CV2. The N3 group had high firing rates, strong attentional modulation (heat map of figure S5), and low Lv (figure S6). The different broad spiking clusters differentiated strongly along dimensions of attentional modulation, Lv, and to some extent FR, and CV2 (figure S6). Similarly narrow spiking clusters exhibited significant differences along dimensions of AUROC, FR, Cv2, and Lv (figure S6). The heat map in figure S5 shows that clusters B3 had very low attROC values. Given that some cells reduced their attend RF activity below the attend away activity (which results in attROC values of <0.5, as described in the maim text), these cells largely comprised the B3 group. Cluster B2 and N2 had fairly moderate attROC values (mostly close to 0.5 in real terms), i.e. fairly weak attentional modulation. Cluster B1 had slightly larger attentional modulation, and cluster B4, N1 and N3 showed strong attentional modulation. The P2T distributions associated with the different clusters are shown to the right of figure S5. The values for FR, Lv, Cv (although not used for clustering) and CV2 are shown in figure S6 along with False discovery rate (FDR) corrected pair wise p-values assessing whether the distributions differed between clusters.

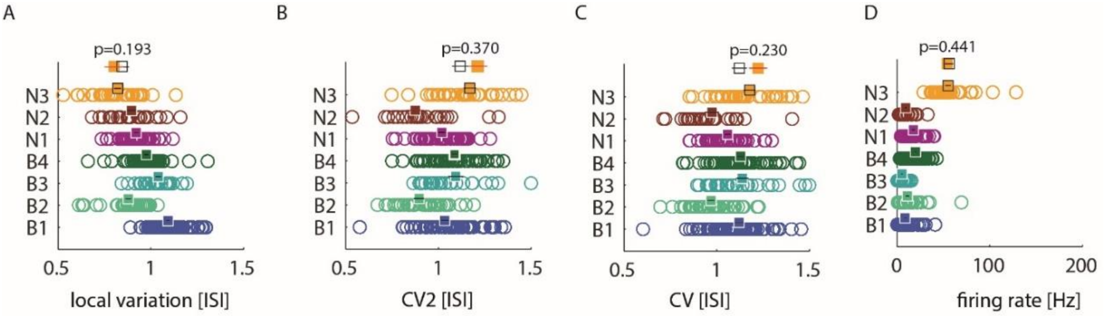

A: F:(6,325=52.1, p<0.001

	B1	B2	B3	B4	N1	N2
B2	0.000					
B3	0.008	0.000				
B4	0.000	0.000	0.002			
N1	0.000	0.044	0.000	0.002		
N2	0.000	0.510	0.000	0.001	0.327	
N3	0.000	0.022	0.000	0.000	0.000	0.017

B: F:(6,325)=12.6, p<0.001

	B1	B2	B3	B4	N1	N2
B2	0.000					
B3	0.185	0.000				
B4	0.090	0.000	0.858			
N1	0.701	0.001	0.097	0.025		
N2	0.001	0.723	0.002	0.000	0.003	
N3	0.001	0.000	0.185	0.033	0.001	0.000

C: F:(6,325)= 8.5, p<0.001

	B1	B2	B3	B4	N1	N2
B2	0.000					
B3	0.799	0.000				
B4	0.826	0.000	0.826			
N1	0.141	0.002	0.040	0.021		
N2	0.007	0.891	0.004	0.000	0.040	
N3	0.219	0.000	0.505	0.219	0.004	0.000

D: F:(6,325)=102.0, p<0.001

	B1	B2	B3	B4	N1	N2
B2	0.169					
B3	0.026	0.022				
B4	0.000	0.000	0.000			
N1	0.000	0.022	0.000	0.299		
N2	0.677	0.508	0.022	0.000	0.001	
N3	0.000	0.000	0.000	0.000	0.000	0.000

Figure S6: Range of cluster feature values associated with the different cell clusters. **A**) Values of local variation of the interspike interval for clusters B1-4 and N1-N3. **B**) Values of CV2 (standard deviation divided by the mean of neighbouring interspike interval differences) for clusters B1-4 and N1-N3. **C**) Values of coefficient of variation of interspike intervals for clusters B1-4 and N1-N3. **D**) Values of firing rates during the pre-dimming period (for the attend RF condition) for clusters B1-4 and N1-N3. Tables beneath each subplot show pairwise p-values indicating whether or not feature value distributions differed between cell clusters (FDR corrected [adjusted] p-values). Above each table is the initial assessment whether differences existed between cell clusters (1 way ANOVA). At the top of each subplot an additional comparison is made for the N3 group, where we subdivided this group into narrow and broad spiking cells according to the P2T width (250us). Here we determined whether any of the features differed for this division (narrow spiking cell means and S.E.M indicated by yellow

symbols, broad spiking cell means and S.E.M indicated by black symbols). The p-values indicate that no differences were found for this subdivision.

Drug modulation at the population level

Here we describe the effects of cholinergic modulation on overall neuronal excitability at the population response level (which manifest as firing rate changes), The main effects of drug application for broad and narrow spiking cells are shown by the normalized population rate histograms displayed in figure S7. Included are cells that were significantly affected by the drug and by attention. A very similar picture emerged, when all cells were included, irrespective of whether they were affected by the drug or attention. Figure S7 shows the activity for the three different time periods of interest, for each of the different drugs used, separately for narrow and broad spiking cells and separated according to the attentional condition. By visual inspection, it becomes clear that ACh application increases the average neuronal activity for broad and narrow spiking cell for both attention conditions, while muscarinic or nicotinic blockade reduced the average neuronal activity in both cell groups, under both attention conditions.

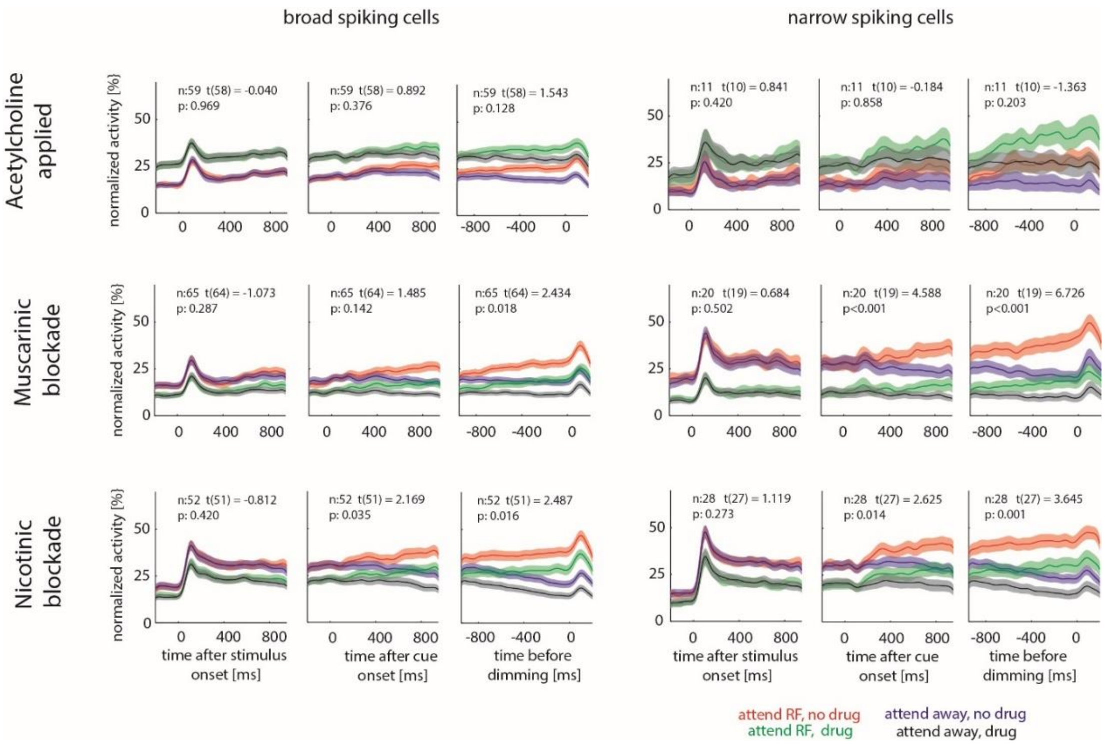

Figure S7: Effect of attention and of drug application on population neuronal firing rates broad and narrow spiking cells, when acetylcholine, scopolamine, or mecamylamine were applied. A) Acetylcholine applied vs. not applied. B) Scopolamine applied vs. not applied. C) Mecamylamine applied vs. not applied. Shaded area shows S.E.M. Red colours: attend RF, no drug applied; Green colours: attend RF, drug applied; Blue colours: attend away, no drug applied; Black colours: attend away, drug applied. Sample sizes are indicated by n, p- values indicate whether drug application affected attention-

induced rate differences. T values give t-statistics of two sided paired t-tests (normalized rate differences drug applied vs. not applied).

Effects of drugs on neuronal firing rates summary statistics

		acetylcholine	scopolamine	mecamylamine
**stimulus period**	**broad**	**p<0.001**<br>**t(1,58)=33.06**	**p<0.001**<br>**t(1,64)=24.32**	**p<0.001**<br>**t(1,61)=23.27**
	**narrow**	**p<0.001**<br>**t(1,10)=18.56**	**p<0.001**<br>**t(1,19)=8.31**	**p<0.001**<br>**t(1,26)=14.01**
**cue period**	**broad**	**p<0.001**<br>**t(1,58)=34.00**	**p<0.001**<br>**t(1,64)=25.80**	**p<0.001**<br>**t(1,61)=23.86**
	**narrow**	**p<0.001**<br>**t(1,10)=18.27**	**p<0.001**<br>**t(1,19)=8.53**	**p<0.001**<br>**t(1,26)=13.01**
**pre dimming period**	**broad**	**p<0.001**<br>**t(1,58)=23.67**	**p<0.001**<br>**t(1,64)=19.75**	**p<0.001**<br>**t(1,61)=20.52**
	**narrow**	**p<0.001**<br>**t(1,10)=31.47**	**p<0.001**<br>**t(1,19)=8.42**	**p<0.001**<br>**t(1,26)=13.02**

SI Appendix Table S2: Summary statistics of drug effects on neuronal firing rates. p-values are FDR adjusted. t-statistics (two side t-test) are given below each FDR adjusted p-value for the three different drugs and the three analysis periods for broad and narrow spiking cells separately.

Difference in firing rates between broad and narrow spiking cells and their potential effects on drug susceptibility

We found a difference in muscarinic blockade efficacy on broad and narrow spiking cell types. it could potentially be the case that this was a consequence of differential firing rates between narrow and broad spiking cells. To test this, we analysed the efficacy of muscarinic blockade on cell excitability for neurons with higher firing rates (irrespective of their cell type assignment) vs. neurons with lower firing rates. Cells were assigned to one of two groups based on the median firing rate in the no drug condition. This split did not result in significantly different drug MIs for the two cell groups (smallest p=0.347, t(1,83)=0.945, two sided t-test).

Drug levels applied and their potential implication on activity changes.

Although drug application was performed with a fairly limited range of drug currents, they varied between experiments (for details see methods). The reason we varied the currents is that in each experiment we aimed to modulate the neuronal activity slightly, without altering it too much. While our previous experience allowed us to restrict our choice of possible application currents, we still varied it based on effects seen in a given session. Specifically, in a given experiment we always monitored the effect of drug application for a few trials, before settling on a level that was then used for the remainder of the day. Thus, the drug application current used for a given cell was fixed, but it could vary between cells. It might thus be the case that the reason why drug effects were seen in some, but not all cells were a consequence of different drug application levels. Moreover, differences seen between different cell types might equally have occurred because drug application currents differed when the respective cell types were recorded.

To address these concerns we first tested whether differences in drug application current could account for whether or not cells showed significant changes in firing rate when drugs were applied. No significant difference in drug application current was found for affected vs. non-affected cells when ACh was applied (affected cells: 24.3+/-15 nA, non-affected cells:19.7+/-10.2 nA, t (1,82)=1.2, p=0.25, two sided t-test). For the sample tested with scopolamine, drug application levels differed significantly between affected and non-affected cells (affected cells:36.8+/-18.9 nA, non-affected cells:25.3+/-15.1 nA, t(1,133)=3.7, p<0.001, two sided t-test). No significant differences in drug current level existed between affected and non-affected cells tested with mecamylamine (affected cells:21.4+/-9.6 nA, non-affected cells:18.6+/-8.7 nA, t(1,120)=1.6, p=0.12, two sided t-test). Thus, for ACh and mecamylamine the amount of drug applied was not different for affected vs. non-affected cells. When scopolamine was applied, we cannot rule out the possibility that the amount of drug applied was a critical factor, which determined whether or not a cell was affected by drug application.

Another, and likely more crucial, concern for the conclusions of the current paper is whether drug application currents (for a given drug, in cells affected by the drug) differed systematically between narrow and broad spiking cells. To address this we compared the currents used for broad vs. narrow spiking cells when ACh, scopolamine, or mecamylamine were applied. None of the comparisons showed significant difference in current used across any of the cell samples (ACh: narrow spiking 21.5+/-14.8 nA, broad spiking: 26.4+/-15.6 nA, t (1,53)=0.5, p=0.65; Scop narrow spiking: 36.8+/-22.0 nA, broad spiking: 37.2+/-17.5 nA, t (1,72)=0.1, p=0.95; Mec narrow spiking: 23.3+/-8.8 nA, broad spiking: 22.1+/-9.1 nA, t (1,66)=1.3, p=0.20, two sided t-test). We can thus exclude the possibility that differential effects of drug application on

overall firing rates or on attentional modulation in narrow vs. broad spiking cells (as reported below) were a consequence of differing levels of drug application.

Drug modulation of cell excitability across different cell clusters

As described in the main text, we found that muscarinic blockade more strongly affected cell excitability in narrow spiking cells than in broad spiking cells. We further analysed whether this was also the case for specific cell clusters. To that extent we quantified the change in cell excitability by calculating drug MIs when ACh, when scopolamine and when mecamylamine were applied. The results are shown in figure S8.

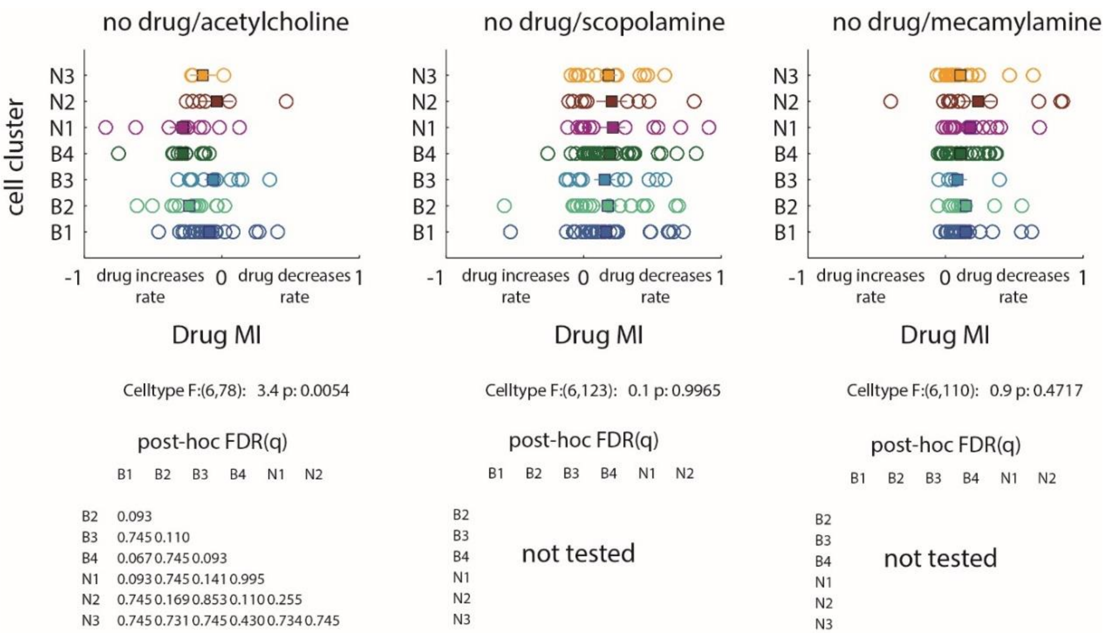

Figure S8: Effect of drug application on neuronal excitability/firing rate. Data points show Drug modulation indices (MI) for the different cell clusters (B1-4 and N1-3). Circles indicate individual drug MIs, square and bars indicate mean and S.E.M (latter usually too small to be visible). Main p-value beneath each subplot indicates whether different clusters are differently affected by the drug application (Mixed-model ANOVA, Main effect of drug on cell-type. Where this was significant, post hoc testing was performed, to determine which clusters differed in their drug susceptibility (p-values are corrected for multiple comparison, FDR adjusted). Based on these adjusted p-values it appears that B1 showed a (mild) trend to differ from B2, B4 and N1. Additionally there was a mild trend for B4 to differ from B3. These trending differences only occurred when Ach was applied. No main effect was found when muscarinic or nicotinic receptors were blocked.

Effect size of drug application on neuronal excitability in different cell types/clusters

We recorded different numbers of cells under the different drugs applied, which was further affected by cluster-analysis. To allow for some quantitative comparison between drug efficacy on altering neuronal excitability, we calculated effect sizes as

$$\theta = \frac{\mu_{firing\ rate\ no\ drug} - \mu_{firing\ rate\ drug}}{\sigma_{(firing\ rate\ no\ drug, drug)}}$$

whereby μ is the mean of the attend RF firing rate distribution given the cell type/cluster studied, and σ is the standard deviation of the attend RF firing rate of the joint distribution.

	**broad**	**narrow**	**B1**	**B2**	**B3**	**B4**	**N1**	**N2**	**N3**
**Acetylcholine**	-0.439	-0.454	-0.237	-0.631	-0.549	-0.897	-0.581	-0.248	-1.557
**Scopolamine**	0.291	0.699	0.428	0.049	0.237	0.629	0.704	0.729	1.091
**Mecamylamine**	0.223	0.318	0.665	0.835	-1.414	0.546	0.534	0.536	0.452

SI Appendix table S3: Effect size of drug application on neuronal activity (excitability).

Drug effects on attentional modulation

As stated in the main text, some cells showed attentional AUROC values <0.5 (i.e. attention reduced neuronal activity relative to attend away conditions). It could be argued that for these cells AUROC values should be calculated as 1-AUROC, and any statistics should be based on populations where this conversion is applied. Doing so did not affect the main conclusions. Specifically, with such conversion, acetylcholine decreased attentional modulation in broad spiking cells [p=0.004] but it had no effect in narrow spiking cells [p=0.594]. Scopolamine decreased attentional modulation in broad and narrow spiking cells [p<0.001, and p=0.002 respectively]. Mecamylamine did not affect attentional modulation in broad spiking cells [p=0.101], but it reduced attentional modulation in narrow spiking cells [p=0.018].

Effect size of drug application on attentional modulation in different cell types/clusters

We recorded different numbers of cells under the different drugs applied. The cluster-analysis further altered (reduced) cell numbers available for statistical comparison. To allow for some quantitative comparison between drug efficacy on altering attentional modulation in samples of different numbers, we calculated effect sizes as

$$\theta = \frac{\mu_{no\ drug} - \mu_{drug}}{\sigma_{(no\ drug, drug)}}$$

whereby μ is the mean of the respective attentional ROCs given the cell type/cluster studied, and σ is the standard deviation of the joint distribution.

	**broad**	**narrow**	**B1**	**B2**	**B3**	**B4**	**N1**	**N2**	**N3**
**Acetylcholine**	0.279	0.175	0.419	0.202	-0.411	0.633	0.022	0.537	0.472
**Scopolamine**	0.165	0.356	0.642	0.164	-0.707	0.681	0.722	0.341	0.425
**Mecamylamine**	0.047	0.411	0.307	-0.489	-1.659	0.262	0.395	0.114	0.444

SI Appendix table S4: Effect size of drug application on attention modulation as measured by AUROCs.

Attentional and drug modulation of firing rate variance

Previous studies have demonstrated that attention reduces firing rate variability in addition to affecting firing rates of neurons in visual cortex [4, 8, 9]. We quantified rate variability by calculating Fano-factors (FF=variance of rate/mean rate) as well as by calculating gain variance [2, 10]. As reported previously [2, 11, 12] attention does not systematically affect FFs in FEF ($F(1,735)=0.42$, $p=0.518$, MM-ANOVA, main effect of attention). Equally, drug application did not have systematic effects on FFs ($F(1,735)=3.735$, $p=0.091$, MM-ANOVA). However, FFs significantly depended on cell type ($F(1,735)=7.66$, $p=0.006$, MM-ANOVA). There were no significant interactions between any of the main effects (all $p>0.3$, MM- ANOVA). The fact that FFs were not affected by drug application or attention, but by cell type, is not surprising. FFs are affected by overall firing rate, due to the non-linear, expansive relationship between mean rate and rate variance [2]. Narrow spiking cells have higher firing rates than broad spiking cells [2], and as a consequence have higher FFs (see MM ANOVA results above). Thus, if blockade of muscarinic receptors increased rate variability, while simultaneously decreasing

firing rate, FFs might not change. Analysis of gain variance [2, 10] is designed to eliminate this problem, and thus this measure has been reported in the main paper.

	broad gains	narrow gains	RF gains	away gains
mean+/- SEM	0.29 +/- 0.02)	0.19 +/- 0.04	0.19 +/- 0.02	0.38 +/- 0.03
t-statistics (FDR adjusted p-values)	t(1,242):-2.326 p: 0.021		t(1,121):-4.583 p< 0.001	

SI Appendix TableS 5: Gain variance in the two main cell types and for the comparison of the attend RF vs. attend away conditions. All data are calculated from 'no drug' trials.

	t-statistics (FDR adjusted p-values)	no Drug gains (all cells)	Drug gains (all cells)
**Acetylcholine**	t(1,71)=0.544 p= 0.728	0.35 +/- 0.04	0.32 +/- 0.06
		**no Drug gains (narrow cells)**	**Drug gains (narrow cells)**
	t(1,9)=0.443 p= 0.728	0.51 +/- 0.20	0.42 +/- 0.23
		**no Drug gains (broad cells)**	**Drug gains (broad cells)**
	t(1,61)=0.349 p= 0.728	0.32 +/- 0.04	0.30 +/- 0.06
**scopolamine**	**t-statistics (FDR adjusted p-values)**	**no Drug gains (all cells)**	**Drug gains (all cells)**
	t(1,81)=-3.300 p= 0.004	0.30 +/- 0.03	0.47 +/- 0.06
		**no Drug gains (narrow cells)**	**Drug gains (narrow cells)**
	t(1,15)=-2.504 p= 0.024	0.19 +/- 0.03	0.59 +/- 0.15
		**no Drug gains (broad cells)**	**Drug gains (broad cells)**
	t(1,65)=-2.315 p= 0.024	0.33 +/- 0.04	0.45 +/- 0.06
**mecamylamine**	**t-statistics (FDR adjusted p-values)**	**no Drug gains (all cells)**	**Drug gains (all cells)**
	t(1,89)=-4.730 p< 0.001	0.16 +/- 0.01	0.28 +/- 0.02
		**no Drug gains (narrow cells)**	**Drug gains (narrow cells)**
	t(1,33)=-3.697 p= 0.001	0.10 +/- 0.01	0.24 +/- 0.04
		**no Drug gains (broad cells)**	**Drug gains (broad cells)**
	t(1,55)=-3.108 p= 0.003	0.20 +/- 0.02	0.30 +/- 0.03

SI Appendix Table S6: Gain variance (mean +/- SEM) as a function of drug application across all cells and separately for the two main cell types. P-values from t-statistics are FDR corrected for multiple comparisons.

Do cholinergic drugs affect attention induced changes to gain variance?

To determine whether drug application altered the effect of attention on gain variance we calculated ($Attention\ Gain\ MI = \frac{Gain\ Var_{attRF} - Gain\ Var_{att\ away}}{Gain\ Var_{attRF} + Gain\ Var_{att\ away}}$) for the control and the drug condition. If a drug altered the effect of attention on gain variance in a systematic manner, we would expect the control and drug attention gain MIs to differ significantly. This was not the case for any of the drugs applied (mm-ANOVAs, all p-values >0.132, SI Appendix fig. S9 for details).

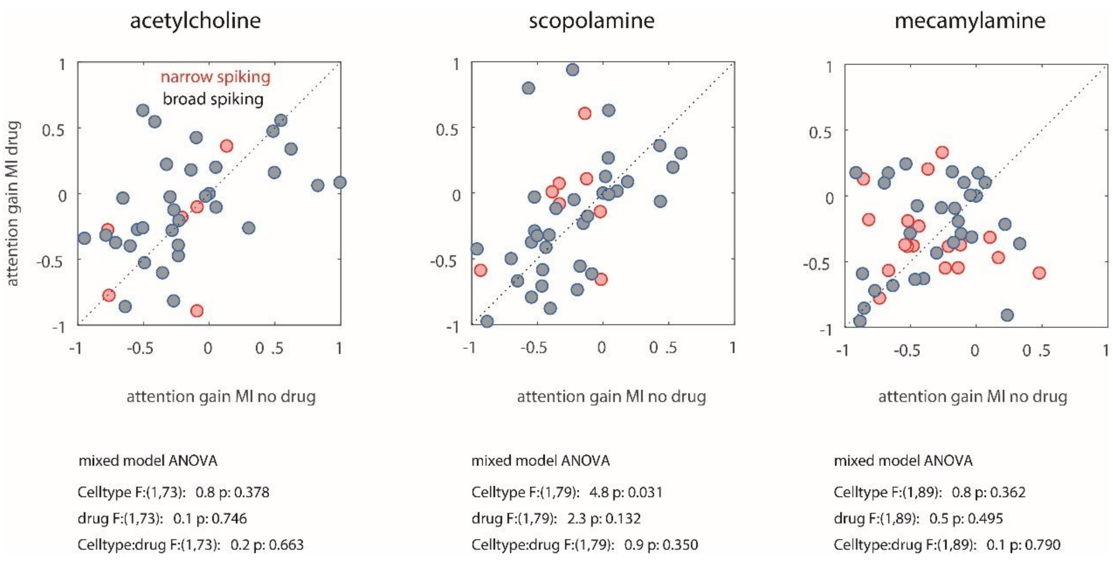

Figure S9: Effect of attention on gain variability with and without drug applied. The effect of attention of gain variability was quantified by calculation a modulation index (MI, results section for details). A mixed model ANOVA was used to determine whether the effect of attention on gain variance differed between cell types and between control conditions vs drug applied conditions.

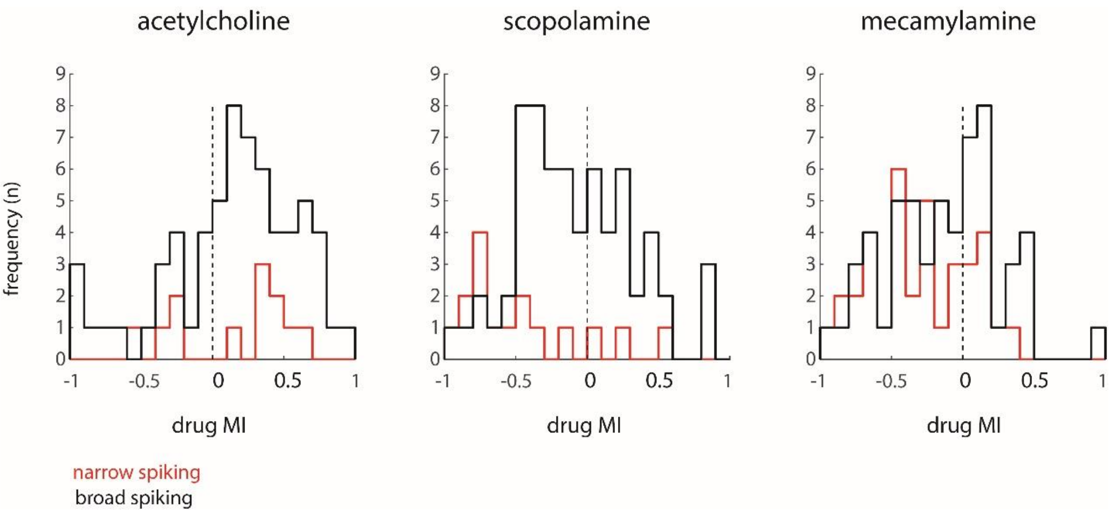

Figure S10: Effect of drug application on gain variability. The effect of drug application of gain variability was quantified by calculation a modulation index (MI, results section for details).

Attentional modulation of gain variance across different cell clusters

A

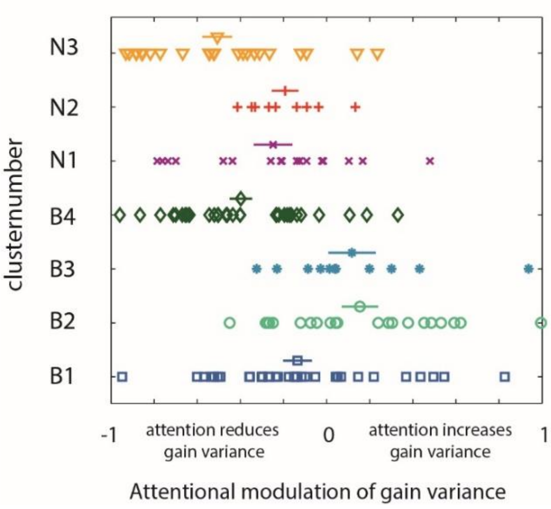

B

mixed model ANOVA

Term	FStat	pValue
(Intercept)	F:(1,128): 32.6	p: 0.0000
Celltype	F:(6,128): 10.1	p: 0.0000
drug	F:(2,128): 1.8	p: 0.1770
Celltype*drug	F:(12,128): 0.6	p: 0.8019

C

post-hoc FDR(q)

	B1	B2	B3	B4	N1	N2
B2	0.032					
B3	0.092	0.558				
B4	0.030	0.000	0.001			
N1	0.515	0.015	0.042	0.593		
N2	0.770	0.032	0.042	0.377	0.842	
N3	0.007	0.000	0.001	0.146	0.064	0.369

Figure S11: Effect of attention on neuronal rate variability as assesses through gain variance. **A**) Data points show attention modulation indices of gain variance for the different cell clusters (B1-4 and N1-3). Individual data points centered on the grouping name indicate individual attentional MIs, data points just above the grouping names (and bars) indicate mean and S.E.M. **B**) Results from a mixed model ANOVA, which determined whether attentional modulations of gain variance differed between cell types (clusters), or whether it was affected by drug application. **C**) Since there were significant differences between cell-clusters, we performed post-hoc testing to determine which groups differed in terms of their attentional modulation of gain variance. P-values are corrected for multiple comparison, FDR adjusted. Attentional modulation of gain variance differed between B4 and B1, B2 and B3, respectively. Moreover, N1 and N2 differed from B2 and B3. Finally, N1 differed from B1, B2, and B3.

Drug effect on reaction times

Reaction times were normalized relative to the session mean, whereby each session mean was calculated across all attention and all drug conditions. This normalization was done to account for differences in receptive field locations and sizes, which affect eccentricity and stimulus size. The latter in turn affect task difficulty and thus reaction times. Effects of attention and of drug application were assessed based on the normalized single trial RTs using a 2 factor ANOVA. Post hoc testing was done based on rank sum tests.

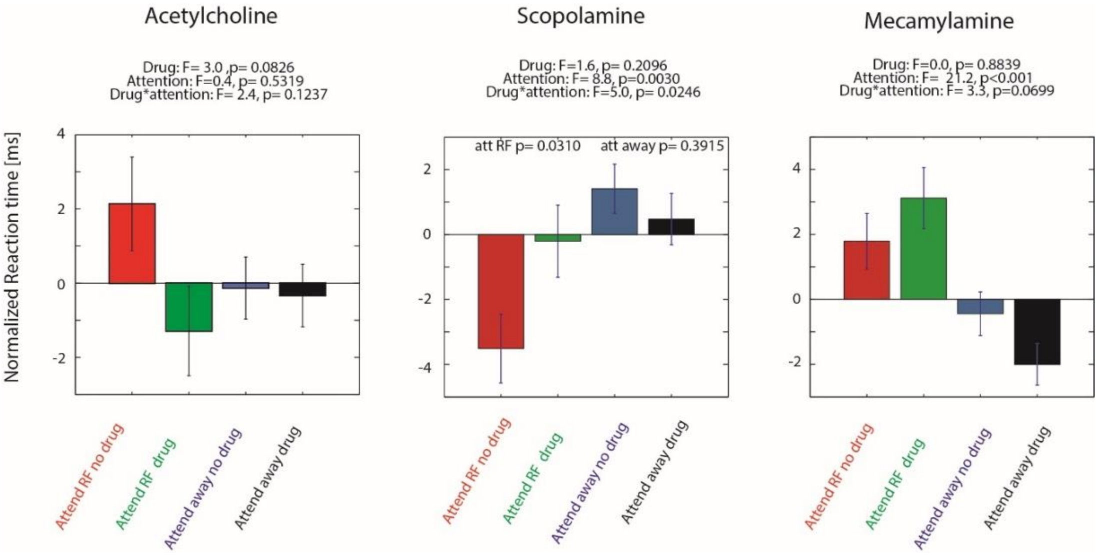

Figure S12: Effect of drug application on reaction times. Shown are normalized reaction times for the attend RF and away condition when no drug was applied (red and blue respectively), and when the drug was locally applied (green and black, respectively). When ACh was applied there was a trend towards reduced reaction times when the animals attended to the RF (compare red and green bars, p-value for drug effect is shown in the ANOVA table above each subplot). When scopolamine was applied there was a significant increase in reaction times only for the attend RF condition (compare red and green bars vs. blue and black bars, p-value for drug effect is shown in the ANOVA table above the subplot, pairwise comparisons are given as insets). No effect on reaction time was found when nicotinic receptors were blocked with mecamylamine.

drug	Attended location	Normalized Reaction times [ms]	ANOVA p-value<br>Drug (D)<br>Attention (A)<br>Interaction (D*A)	Post-hoc p-value
Agonist ACh	RF no drug	2.13+/-1.27	p(D)=0.082<br>p(A)=0.531<br>p(D*A)=0.123	0.051
	RF drug	-1.29+/-1.21		
	away no drug	-0.13+/-0.84		0.863
	away drug	-0.33+/-0.84		
Muscarinic blockade	RF no drug	-3.51+/-1.06	p(D)=0.209<br>p(A)=0.002<br>p(D*A)=0.025	0.031
	RF drug	-0.20+/-1.11		
	away no drug	1.41+/-0.76		0.315
	away drug	0.47+/-0.79		
Nicotinic blockade	RF no drug	1.78+/-0.85	p(D)=0.884<br>p(A)<0.001<br>p(D*A)=0.069	0.296
	RF drug	3.11+/-0.94		
	away no drug	-0.44+/-0.67		0.093
	away drug	-2.00+/-0.67		

SI Appendix Table S7: Effect of drug application on behavioural reaction times. Reaction times are calculated relative to each session mean.

SI Appendix References

1. Gray, H., et al., *Physiological, Behavioral, and Scientific Impact of Different Fluid Control Protocols in the Rhesus Macaque (Macaca mulatta).* eNeuro, 2016. **3**(4).
2. Thiele, A., et al., *Attention Induced Gain Stabilization in Broad and Narrow-Spiking Cells in the Frontal Eye-Field of Macaque Monkeys.* J Neurosci, 2016. **36**(29): p. 7601-12.
3. Thiele, A., et al., *A novel electrode-pipette design for simultaneous recording of extracellular spikes and iontophoretic drug application in awake behaving monkeys.* J Neurosci Methods, 2006. **158**(2): p. 207-11.
4. Mitchell, J.F., K.A. Sundberg, and J.H. Reynolds, *Differential attention-dependent response modulation across cell classes in macaque visual area V4.* Neuron, 2007. **55**(1): p. 131-41.
5. Ardid, S., et al., *Mapping of functionally characterized cell classes onto canonical circuit operations in primate prefrontal cortex.* J Neurosci, 2015. **35**(7): p. 2975-91.
6. Holt, G.R., et al., *Comparison of discharge variability in vitro and in vivo in cat visual cortex neurons.* J Neurophysiol, 1996. **75**(5): p. 1806-14.
7. Shinomoto, S., K. Shima, and J. Tanji, *Differences in spiking patterns among cortical neurons.* Neural Comput, 2003. **15**(12): p. 2823-42.
8. Herrero, J.L., et al., *Attention-induced variance and noise correlation reduction in macaque V1 is mediated by NMDA receptors.* Neuron, 2013. **78**(4): p. 729-39.
9. Cohen, M.R. and J.H. Maunsell, *Attention improves performance primarily by reducing interneuronal correlations.* Nat Neurosci, 2009. **12**(12): p. 1594-600.
10. Goris, R.L., J.A. Movshon, and E.P. Simoncelli, *Partitioning neuronal variability.* Nat Neurosci, 2014. **17**(6): p. 858-65.
11. Chang, M.H., K.M. Armstrong, and T. Moore, *Dissociation of response variability from firing rate effects in frontal eye field neurons during visual stimulation, working memory, and attention.* J Neurosci, 2012. **32**(6): p. 2204-16.
12. Purcell, B.A., et al., *Response variability of frontal eye field neurons modulates with sensory input and saccade preparation but not visual search salience.* J Neurophysiol, 2012. **108**(10): p. 2737-50.
